# From Kinetics to Molecular-Level Insights into Group 4 Metal Oxide Nanocrystal Synthesis

**DOI:** 10.1021/acsmaterialsau.5c00032

**Published:** 2025-05-29

**Authors:** Carlotta Seno, Christopher B. Whitehead, David E. Salazar Marcano, Ian Chaon, Jonathan De Roo

**Affiliations:** † Department of Chemistry, 27209 University of Basel, Mattenstrasse 22, 4058 Basel, Switzerland; ‡ Department of Chemistry, 7254 Union College, Schenectady, New York 12305, United States

**Keywords:** mechanism, COPASI, titanium, zirconium, hafnium, nanoparticle

## Abstract

Kinetic control is a powerful tool for traversing the chemical landscape toward the intended product. For group 4 metal oxide nanocrystals, the development of complex multimetallic heterostructures is still a challenge, partly due to the lack of kinetic and mechanistic understanding. Here, we study the reaction kinetics of the nonaqueous synthesis of titanium, zirconium, and hafnium oxide nanocrystals, from the decomposition of metal isopropoxide and metal halide, in the presence of tri-*n*-octylphosphine oxide (TOPO). The reaction rate depends on the metal: Ti ≫ Zr > Hf. While titanium follows an S_N_1 substitution mechanism, zirconium and hafnium follow an auto-catalyzed E1 elimination. In both cases, the reaction kinetics can be tuned by varying the amount of TOPO or the chloride content due to their impact on the electronic structure of the transition state of the rate-determining step. The proposed mechanism was shown to be consistent with kinetic modeling of the data for different metal concentrations. This deeper understanding of group 4 metal oxide nanocrystal formation will facilitate access to novel heterostructures relevant for optical, catalytic, and electronic materials.

## Graphical abstract

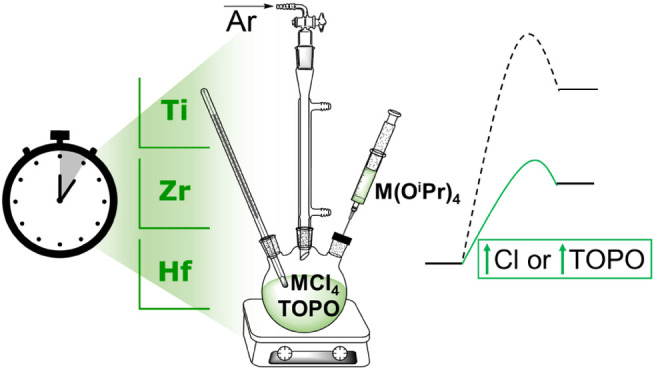

## Introduction

Metal oxides of group 4 (titania, zirconia, and hafnia) form an interesting class of nanomaterials. Titania nanocrystals have applications in medicine, photocatalysis, and photovoltaics.^1–5^ Zirconia nanocrystals are components for (in)-organic composites.^1^ Hafnia nanocrystals have found use as resistive switching elements,^6,7^ as scintillators,^8^ as computed tomography contrast agents,^1,9,10^ and as sensitizers in radiation therapy.^1,11^ All three oxides (but zirconia and hafnia especially) have high thermal and chemical stability, making them attractive for a wide range of applications from nanoelectronics to medicine.^12–14^

Highly monodisperse and colloidally stable oxide nanocrystals can be obtained via nonaqueous surfactant-assisted syntheses.^1^ A particularly successful strategy is the reaction of metal alkoxide with metal halide in tri-*n*-octylphosphine oxide (TOPO).^1,15–17^ Anatase titania is thus produced at 300 °C, while tetragonal zirconia is formed at 340 °C, and monoclinic hafnia is formed at 340–360 °C. The surface of the obtained nanocrystals is covered by a mixture of protonated TOPO, dioctyl phosphinate, and dioctyl pyrophosphonate.^18^ Control over the final properties of the nanocrystals is highly sought after: shape,^19,20^ size,^21–23^ doping,^24^ and crystal structure.^24^ However, so far, achieving such synthetic control remains a challenge. For instance, the ZrO_2_ nanocrystal size can currently only be tuned between 3 and 5.5 nm.^16,21,23^

Nanocrystal heterostructures, such as core/shells or alloys, have attracted increasing interest.^25–27^ For example, mixed-phase TiO_2_/ZrO_2_ nanoparticles were recently shown to display better electrocatalytic activity toward hydrogen evolution than the constituent metal oxides on their own, making them interesting materials for hydrogen fuel cells.^28,29^ Alloyed Hf_*x*_Zr_1–*x*_O_2_ nanocrystals have also been synthesized in TOPO by varying the amount of zirconium and hafnium precursors, yet, the composition of the nanocrystals was always more zirconium-rich than the composition of the precursor mixture. ZrTiO_4_ and HfTiO_4_ remain inaccessible.^17^ From these reports on group 4 metal oxide alloys, it has been proposed that the reactivity of the metals can be ranked as Ti ≫ Zr > Hf, but direct evidence is still lacking.^1^ While core/shell structures have been extensively explored for other nanocrystals, the design of core/shell heterostructures of group 4 metal oxides has lagged behind. Recently, we reported epitaxial ZrO_2_/HfO_2_ core/shell nanocrystals using nonaqueous surfactant-assisted synthesis.^30^ Shelling europium-doped zirconia nanocrystals with pure zirconia significantly increased the photoluminescence lifetime of europium.^30^ While exciting, issues with independent nucleation of shelling material remain a problem, as the factors affecting nucleation or shell growth are poorly understood. To rationally develop heterostructures by design, a better understanding of the reaction kinetics and the reaction mechanism is required. Indeed, the reaction rate has a direct impact on the type of heterostructures that can be produced.^31^

In 1996, Arnal and co-workers^32^ studied the reaction between TiCl_4_ and Ti­(O*^i^* Pr)_4_ at 100 °C, which forms an amorphous gel (there was no surfactant added). Isopropyl chloride was found to be the only coproduct of the reaction (eq 1).

1 Ti−Cl+PriO−Ti→Ti−O−Ti+PrCli

The precursor decomposition followed a sigmoidal shape, suggesting an auto-catalytic mechanism. In addition, the reaction rate depended on the ratio of TiCl_4_ and Ti­(O*^i^* Pr)_4_, with higher reaction rates (and shorter induction times) for higher contents of TiCl_4_. Later, when titania nanocrystals were grown in TOPO at 300 °C from titanium halides and various titanium alkoxides,^15^ the reaction rate was reported to increase with the branching of the alkoxide (Me < Et <*^i^* Pr <^*t*^Bu), consistent with an S_N_1 (or E1) mechanism.

Recently, we studied zirconia synthesis in TOPO.^21,23,33^ When ZrCl_4_ and Zr­(O*^i^* Pr)_4_·*^i^* PrOH are mixed in an equimolar ratio, the metal complexes exchange ligands and form ZrCl_2_(O*^i^* Pr)_2_(TOPO)_2_. The six-fold coordination is completed by two TOPO (Lewis base) ligands. During the decomposition of this precursor towards ZrO_2_, we find ZrCl_3_(O*^i^* Pr)­(TOPO)_2_ as a transient intermediate and, finally, ZrCl_4_(TOPO)_2_ as a reaction coproduct.^21^ Detecting propene as the main volatile coproduct, we proposed a dominant E1 elimination mechanism, yielding Zr–OH moieties. The latter condenses with zirconium isopropoxide, forming Zr–O–Zr bridges and eliminating isopropanol. Given that the precursor decomposition rate is fast with respect to the crystallization rate, the condensation reaction yields small amorphous particles that recrystallize (and grow) on a slower time scale.^23^

Here, we used ^1^H NMR spectroscopy to monitor the decomposition kinetics of the precursor formed after mixing MCl_4_ with M­(O*^i^* Pr)_4_ for the three metals: Ti, Zr, and Hf. This allowed for a direct comparison of the reactivity of the three metals under the same reaction conditions. Furthermore, the temperature, the ratio of MCl_4_ to M­(O*^i^* Pr)_4_, the equivalents of TOPO, and the metal precursor concentration were varied to gain insights into the reaction mechanism and the transition state of the rate-determining step. The kinetics were fitted either using analytical solutions in IGOR or by solving the differential equations numerically in COPASI.

## Results and Discussion

We quantified the kinetics of precursor decomposition for the reaction between the metal halide and metal alkoxide in TOPO, using the disappearance of the isopropoxide resonance in ^1^H NMR spectroscopy as a convenient handle (Figures S1–S3). The original procedures^15–17^ were slightly modified to improve the precision and extent of data acquisition and to have comparable conditions for all three metals (Ti, Zr, Hf). Since TOPO is not an innocent solvent^34^ but also coordinates the precursors^33^ and binds to the nanocrystal surface,^35^ we chose *n*-octadecane (not 1-octadecene due to issues with polymerization^36^) as an inert solvent with a high boiling point (317 °C).^37^ A precise number of TOPO equivalents was added to the reaction mixture, thus decoupling the ligand concentration from the precursor concentration. To have a well-defined start of the reaction, we injected the isopropoxide precursor into a mixture of metal chloride, ligand, and solvent at the reaction temperature.

First, we considered the reaction with equimolar amounts of metal chloride and metal isopropoxide at 275 °C, with 2 equiv of TOPO (with respect to the total metal content); see Figure 1. Since we know from earlier work^1,21,33,38,39^ that the metal halide and metal isopropoxide form the mixed alkoxy chloride, we write the chemical equation succinctly, with this complex as reagent (the reaction scheme also omits the coordinated isopropanol molecule to the metal isopropoxide in case of zirconium and hafnium isopropoxide since it does not take part in the reaction^21^). Qualitatively, the disappearance of the isopropoxide signal is fastest for titania, slower for zirconia, and slowest for hafnia. For zirconia and hafnia, it is clear that the decomposition starts slowly and subsequently accelerates, suggesting an auto-catalytic mechanism.^40–43^ In contrast, for titania, the decomposition appears to be linear. To quantify the kinetics, we fitted titania data with a linear function (Figure S4) and zirconia and hafnia data (Figures S5 and S6) to the Finke–Watzky (FW) two-step model:^44^

2 $$A\overset{k_{1}\;}{\to }B$$

3 $$A+B\overset{k_{2}\;}{\to }2B$$

The equations are pseudo-elementary, which means they do not represent the actual molecular mechanism but are rather a simplification of the many elementary steps in the process.^45^ The corresponding expression for the precursor decomposition is

4 $$\left[A\right]=\frac{k_{1}/k_{2}+{\left[A\right]}_{0}}{1+\frac{k_{1}}{k_{2}{\left[A\right]}_{0}}e^{\left(k_{1}+k_{2}{\left[A\right]}_{0}\right)t}}$$

where [A] is the precursor concentration, which we take here as the isopropoxide concentration, and *t* is the reaction time. We report the rate constants *k*_1_ and *k*_2_ in Table 1 along with the reaction half-life and the reaction end-point. The latter values are useful in the design of syntheses. The error in *k*_1_ is quite high (which is consistent with the literature^46,47^ ), and there is no statistically significant difference in *k*_1_ between zirconia and hafnia. However, zirconia has a *k*_2_ that is double compared to hafnia. The initial rate is one order of magnitude higher for titania compared to zirconia and hafnia. The decomposition of titanium isopropoxide goes to completion in just 3 min, which is 6 times faster than for Zr and 10 times faster than for Hf. Zirconium and hafnium are often considered twin metals with highly similar properties. Here, we find that they do indeed follow the same trend but that hafnium shows slower kinetics.

**Figure 1:**
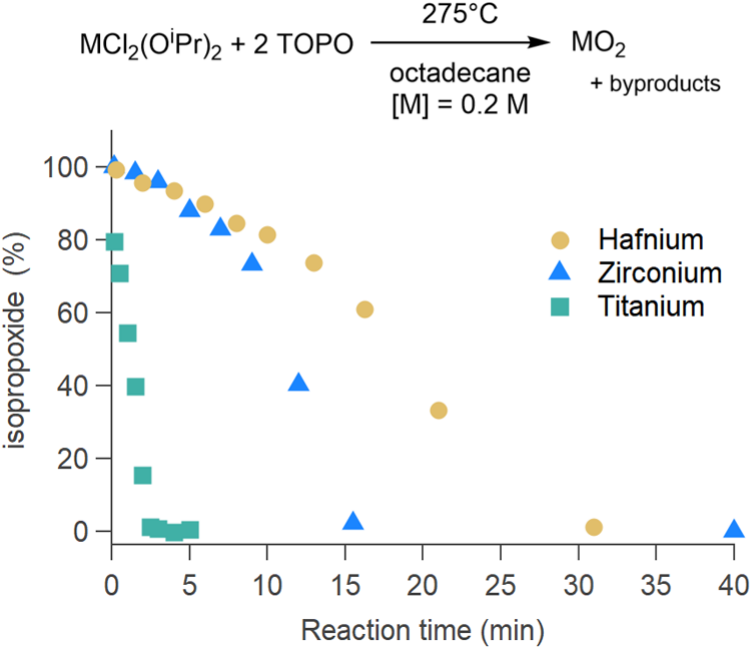
Precursor decomposition, i.e., the disappearance of the isopropoxide resonance in ^1^H NMR (Figures S1–S3) for the reaction of metal chloride and metal isopropoxide (M = Ti, Zr, or Hf).

**Table 1:** Quantification of the Decomposition Kinetics (at 275 °C with 2 TOPO Equivalents), Displayed in Figure 1, and Rate Constants Extracted Using Equation 4

	*k*_1_ (s^–1^)	*k*_2_ (L mol^–1^ s^–1^)	initial rate (mol L^–1^ s^–1^)	half-life (min)	end-point (min)
TiCl_2_(O*^i^* Pr)_2_			2 × 10^–3^	1.5	3
ZrCl_2_(O*^i^* Pr)_2_	(3 ± 2) × 10^–5^	0.022 ± 0.003	1 × 10^–4^	11	18
HfCl_2_(O*^i^* Pr)_2_	(7 ± 2) × 10^–5^	0.010 ± 0.001	1 × 10^–4^	18	31

To uncover more details of the reaction mechanism, we first focused on titania and varied certain reaction parameters. As expected, the reaction rate increases with increasing temperature within the range 250–300 °C (Figure S7A). Given that the kinetics of titanium at 275 °C were too fast to be practical, we focused on collecting kinetics data at 250 °C. We varied the amount of TOPO, taking either 2, 4, or 11.4 equiv (the latter is a synthesis in pure TOPO without octadecane as a co-solvent); see Figure 2B. While the data for 2 equiv is well described by a straight line (apparent zero order kinetics), the data in pure TOPO (11.4 equiv) is perfectly fitted with a single exponential (first-order kinetics). The data for 4 equiv is intermediate and are not well described by either function. We thus analyzed the initial rate, which increases with the TOPO amount (Table 2). This can be rationalized by considering the transition state of the first step in an E1/S_N_1 mechanism (formation of the carbocation). According to the Hammond postulate, the transition state of such an endothermic reaction should reflect the structure of the products. Given that the reaction solvent (octadecane) is highly nonpolar, the buildup of charge is unfavorable. By adding the polar Lewis base (TOPO), the transition state and the final carbocation are stabilized; see Scheme 1, resulting in a faster initial rate with increasing amounts of TOPO. While it is generally acknowledged that ligands have an influence on nanocrystal growth rates, here the impact of the phosphine oxide on the precursor decomposition is demonstrated.

**Figure 2:**
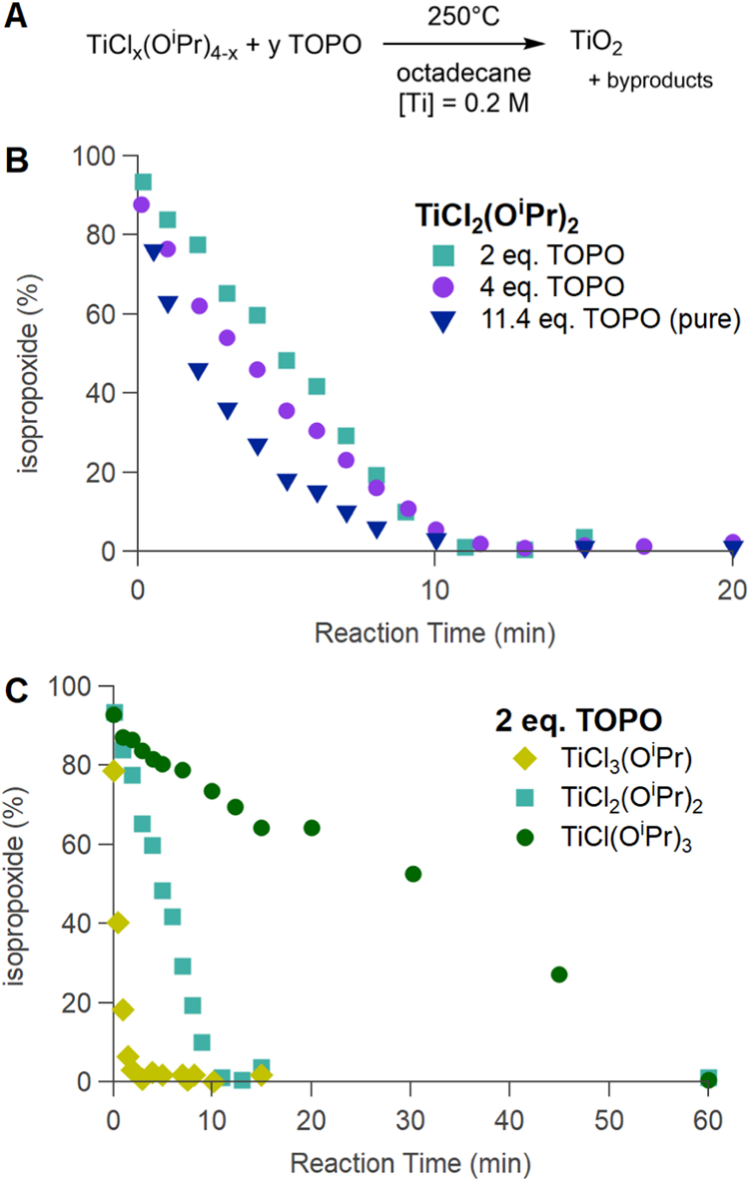
(A) Precursor decomposition for titania at 250 °C, either varying (B) the amount of the Lewis base (TOPO) or (C) the ratio of chloride to isopropoxide in the starting compound.

**Scheme 1:**
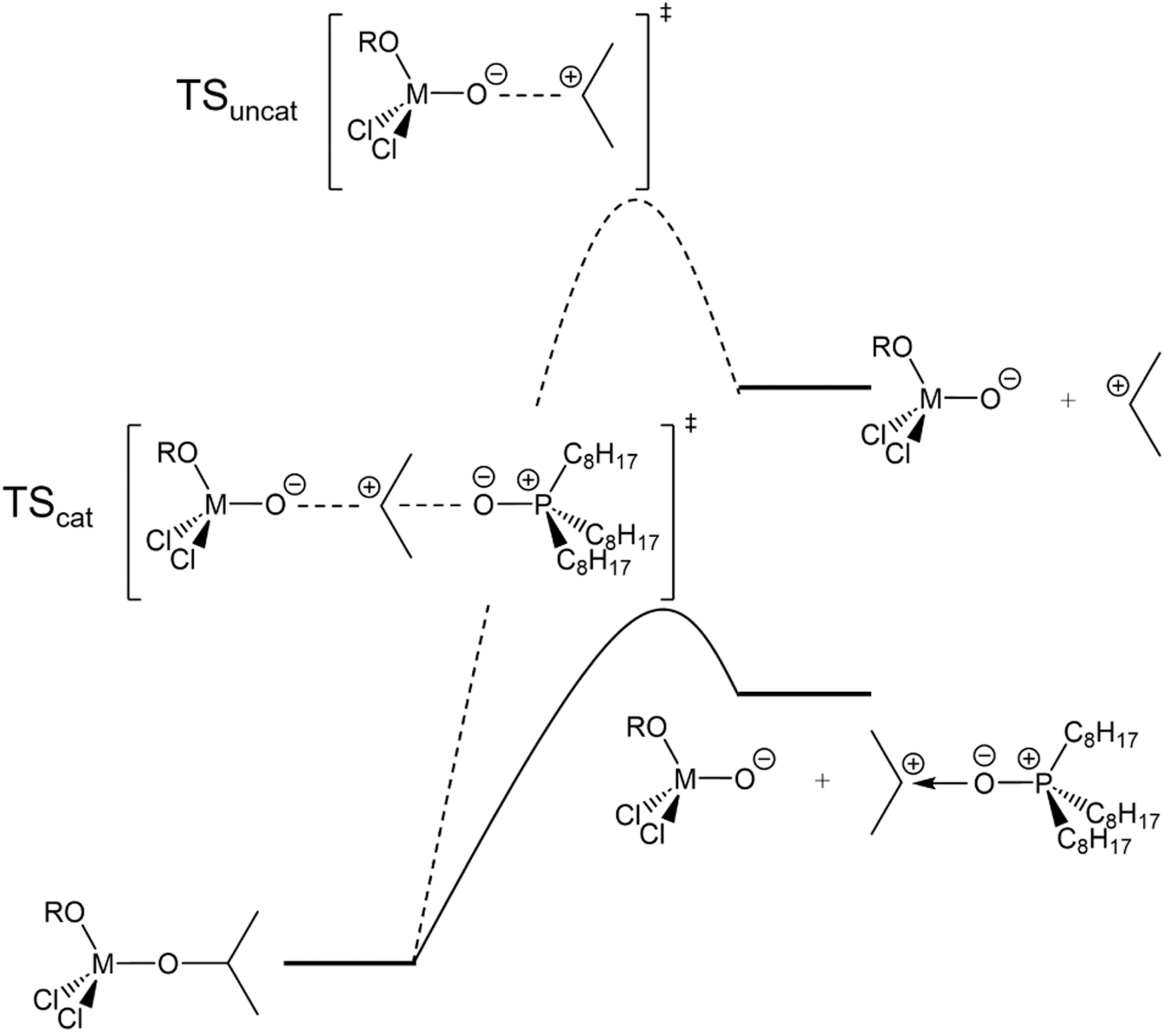
Catalyzed (TS_cat_) and Uncatalyzed (TS_uncat_) Transition State for the Isopropoxide Decomposition^a^

a This is the first and rate-limiting step in the S_N_1/E1 mechanism. The carbocation and the transition state are stabilized by the polar Lewis base.

**Table 2:** Quantification of the Decomposition Kinetics for the Titania Case, Displayed in Figure 2

		initial rate (mol L^–1^ s^–1^)
TOPO	2 equiv	(6 ± 1) × 10^–4^
	4 equiv	(9 ± 0.1) × 10^–4^
	11.4 equiv	(13 ± 1) × 10^–4^
Cl:OR	TiCl_3_(O*^i^* Pr)	(20 ± 5) × 10^–4^
	TiCl_2_(O*^i^* Pr)_2_	(6 ± 1) × 10^–4^
	TiCl(O*^i^* Pr)_3_	(3 ± 2) × 10^–4^

We also varied the titanium chloride-to-isopropoxide ratio from 3:1 to 1:3 while keeping the overall titanium concentration at 0.2 mol/L (Figure 2C). After ligand scrambling, this yields TiCl_3_(O*^i^* Pr), TiCl_2_(O*^i^* Pr)_2_, and TiCl­(O*^i^* Pr)_3_ as reagents. The higher chloride content not only accelerates the reaction but also changes the shape of the decomposition trace. For TiCl_3_(O*^i^* Pr), the decomposition is described well by a single exponential (first-order kinetics), as shown in Figure S8. For TiCl_2_(O*^i^* Pr)_2_ and TiCl­(O*^i^* Pr)_3_, a linear fit is most adequate (Figures S9–S12). An initial rate analysis (Table 2) shows that the decomposition rate of TiCl_3_(O*^i^* Pr) is about 10 times faster than that of TiCl­(O*^i^* Pr)_3_. We can rationalize this again in Scheme 1. The transition state is stabilized by either stabilizing the positive charge on the carbocation or the negative charge on the leaving group (the titanium oxide complex). Replacing an electron-donating isopropoxide with an electron-withdrawing chloride makes the titanium more Lewis acidic, thus stabilizing the negative charge and accelerating the reaction. Therefore, varying the ratio of chloride to isopropoxide offers an alternative strategy for tuning the reaction kinetics.

Based on the kinetics, one cannot distinguish an E1 elimination from an S_N_1 nucleophilic substitution, since they have the same rate-limiting step: the formation of the carbocation. However, the products are different. While propene is formed through elimination, isopropyl chloride is formed through nucleophilic substitution. At 100 °C, in the absence of TOPO, isopropyl chloride had been detected by Arnal and co-workers.^32^ Here, we sampled the headspace of the decomposition of TiCl_2_(O*^i^* Pr)_2_ at 250 °C with 2 equiv of TOPO and analyzed the gaseous products quantitatively with gas chromatography (Figure 3). We found that *^i^* PrCl is formed at a similar rate as the precursor decomposition, confirming the S_N_1 mechanism. Indeed, by fitting a linear function to the first three data points, the slopes are of the same order of magnitude: −3.7 × 10^–5^ for the decomposition of the isopropoxide and 2.0 × 10^–5^ for the formation of isopropyl chloride. In a second step, *^i^* PrCl decomposes into propene and HCl.

**Figure 3:**
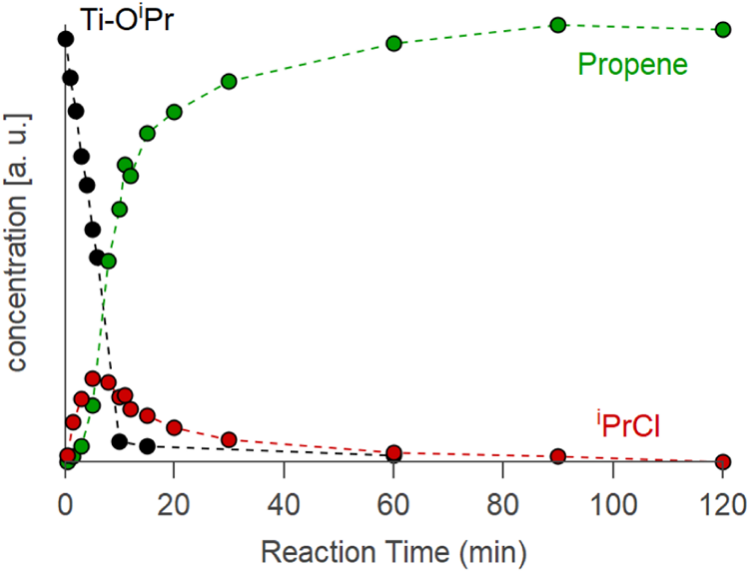
Normalized concentration of propene and isopropyl chloride in the reaction headspace monitored with GC-FID and of isopropoxide followed by ^1^H NMR, for TiO_2_ formation at 250 °C with 2 equiv of TOPO.

We varied the titanium concentration in the presence of 11.4 equiv of TOPO at 250 °C. For 0.1, 0.2, and 0.3 mol/L of TiCl_2_(O*^i^* Pr)_2_, all decomposition traces appear to follow first-order kinetics (Figures S13–S14), and we fitted them with the following first-order reaction

$${\mathrm{TiCl}}_{2}(O^{i}\mathrm{Pr}{)}_{2}\overset{k\;}{\to }{\mathrm{TiO}}_{2}+2^{i}\mathrm{PrCl}$$ 5

which is now a fully balanced equation, although still a pseudo-elementary step and the actual mechanism involves several complex Ti–O–Ti condensation steps. However, the rate-determining step is the S_N_1 step. The first-order rate constants were 0.0047 ± 9 × 10^–5^, 0.0044 ± 1 × 10^–4^, and 0.0036 ± 6 × 10^–5^ s^–1^ for [Ti] = 0.1, 0.2, and 0.3 mol/L, respectively. The rate constant is thus independent of the concentration, further strengthening our mechanistic proposal.

Next, we turned our attention to zirconia. Consistent sigmoidal decomposition kinetics were observed under all reaction conditions investigated. Therefore, we fitted the data with the FW model, thus extracting *k*_1_ and *k*_2_. With increasing temperature, the induction delay visibly shortens (Figure S7B), and both rate constants increase, following Arrhenius-type behavior (see Table S4 and Figures S15 and S16). At 300 °C, we varied the amount of TOPO, taking either 2, 4, 6, or 11.4 equiv; see Figure 4B and Table 3.

**Figure 4:**
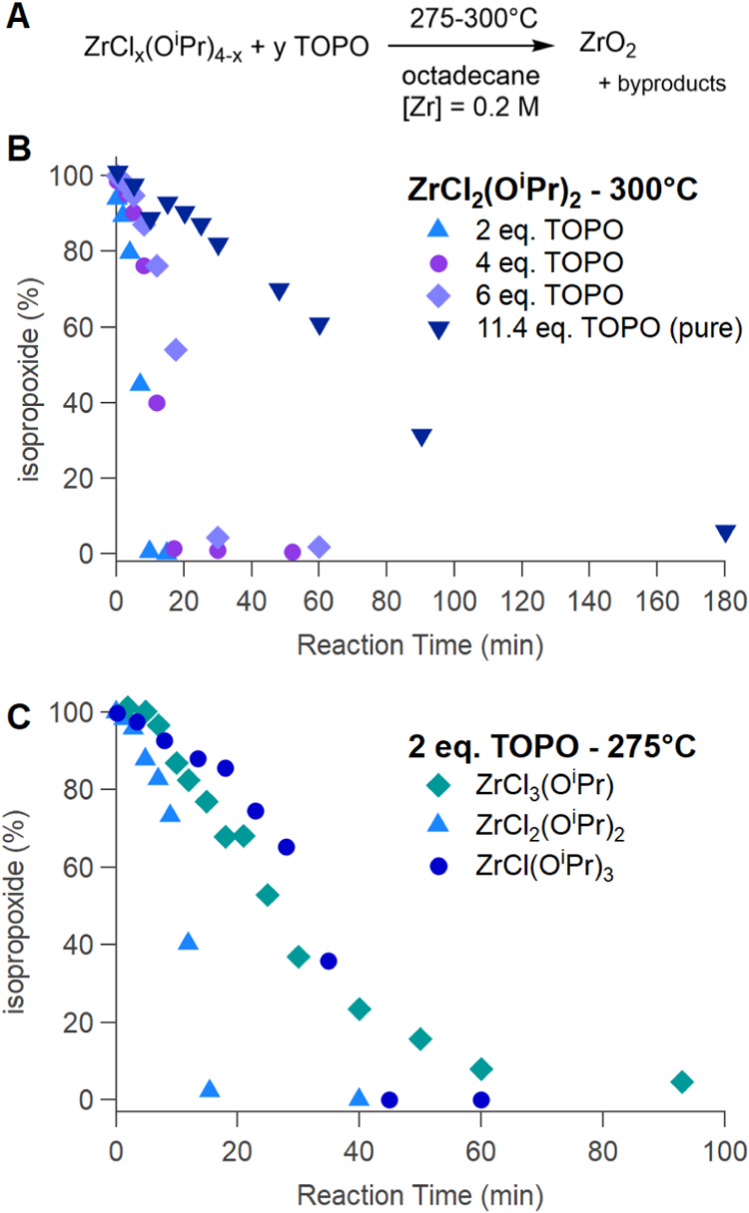
(A) Precursor decomposition for zirconia, either varying (B) the amount of the Lewis base (TOPO) or (C) the ratio of chloride to isopropoxide in the starting compound.

**Table 3:** Quantification of the Decomposition Kinetics for Zirconium Displayed in Figure 4 and Fitting According to the Two-Step FW Model

		*k*_1_ (s^–1^)	*k*_2_ (L mol^–1^ s^–1^)
TOPO	2 equiv	(4 ± 5) × 10^–5^	(40 ± 10) × 10^–3^
	4 equiv	(6 ± 2) × 10^–5^	(20 ± 2) × 10^–3^
	6 equiv	(9 ± 3) × 10^–5^	(10 ± 1) × 10^–3^
	11.4 equiv	(7 ± 1) × 10^–5^	(1 ± 0.1) × 10^–3^
Cl/OR	ZrCl_3_(O*^i^* Pr)	(30 ± 7) × 10^–5^	(6 ± 0.6) × 10^–3^
	ZrCl_2_(O*^i^* Pr)_2_	(3 ± 2) × 10^–5^	(20 ± 3) × 10^–3^
	ZrCl(O*^i^* Pr)_3_	(1 ± 0.7) × 10^–5^	(5 ± 0.8) × 10^–3^

Although one expects the same stabilizing effect of TOPO on the transition state for the decomposition of zirconium isopropoxide as observed for titanium (Scheme 1), this should only affect *k*_1_ since *k*_2_ reflects the auto-catalysis by a reaction product. While there appears to be the expected trend for *k*_1_ in the formation of zirconia (Table 3), the large errors in the fitted values do not allow for a strong conclusion. This is likely because the effect of TOPO on *k*_1_ is small, as it is also the case for titania: the initial rate only increased by a factor of 2 when the amount of TOPO was increased from 2 to 11.4 equiv. Nevertheless, in stark contrast to titania, TOPO clearly slows down the overall reaction rate, and this is reflected in a decreasing *k*_2_ with the TOPO amount; see Figure 5 (note the logarithmic *y*-axis).

**Figure 5:**
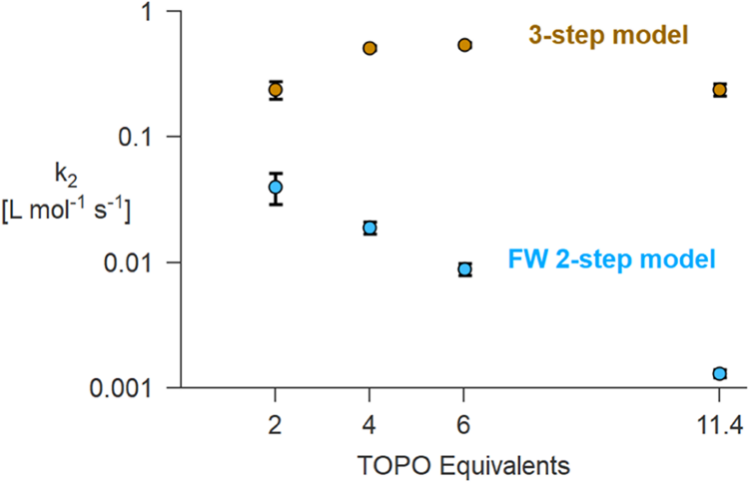
Values of the *k*_2_ rate constant obtained by fitting the zirconia kinetics with different amounts of TOPO at 300 °C; see Figure 4B. The fits were performed with the two-step FW model and alternatively with the three-step model that adds an association equilibrium between the catalyst and TOPO.

We infer that TOPO binds to the product that is capable of auto-catalysis. We thus adapted the FW 2-step model by adding a third step where the catalyzing reaction product (B) reversibly binds to TOPO.^48^

6 $$A\overset{k_{1}\;}{\to }B$$

7 $$A+B\overset{k_{2}\;}{\to }2B$$

8 $$B+2\mathrm{TOPO}\overset{K_{eq}\;}{⇋}B(\mathrm{TOPO}{)}_{2}$$

As this set of equations becomes too difficult to solve analytically, the differential rate equations were solved numerically by COPASI. We explored various stoichiometries and equilibrium constants and found that a stoichiometry of 2 with *K*_eq_ = 12 minimizes the variation in the second rate constant (*k*_2_). Whereas the two-step model causes a trend in *k*_2_ that spans more than an order of magnitude, the three-step model reduces the variation to twofold; see Figure 5. This suggests that the slower precursor decomposition with increasing TOPO concentration is due to an equilibrium that reduces the availability of the auto-catalytic species, such as the formation of a complex with TOPO. Unlike for titania, this auto-catalytic step dominates the overall rate of the reaction.

When varying the zirconium chloride-to-isopropoxide ratio (Figure 4C and Table 3), we find that with increasing chloride content in the precursor, *k*_1_ increases, meaning that the non-catalyzed reaction is faster with more chloride. This is similar to the titania case and likely has the same origin (Scheme 1). On the other hand, the auto-catalytic rate constant *k*_2_ does not follow a clear trend and is highest for ZrCl_2_(O*^i^* Pr)_2_.

We previously established that zirconia is predominantly formed through an E1 elimination, with a small contribution from S_N_1 substitution.^21^ As a balanced mechanism, we thus combine an E1 elimination reaction with a condensation and an auto-catalytic step (Figure 6). In this mechanism, zirconium isopropoxide is consumed through both E1 elimination and condensation steps. The equations are fully balanced and take into account the presence of ZrCl_4_ as the reaction coproduct previously observed by ^31^P NMR.^21^ The concentration of ZrCl_2_(O*^i^* Pr)_2_ in the presence of 11.4 equiv of TOPO at 340 °C was changed from 0.1, 0.2, and 0.3 mol/L (Figure S17), and the differential rate equations were solved numerically by COPASI. A highly satisfactory fit was obtained in all three cases (Figure 6).

**Figure 6:**
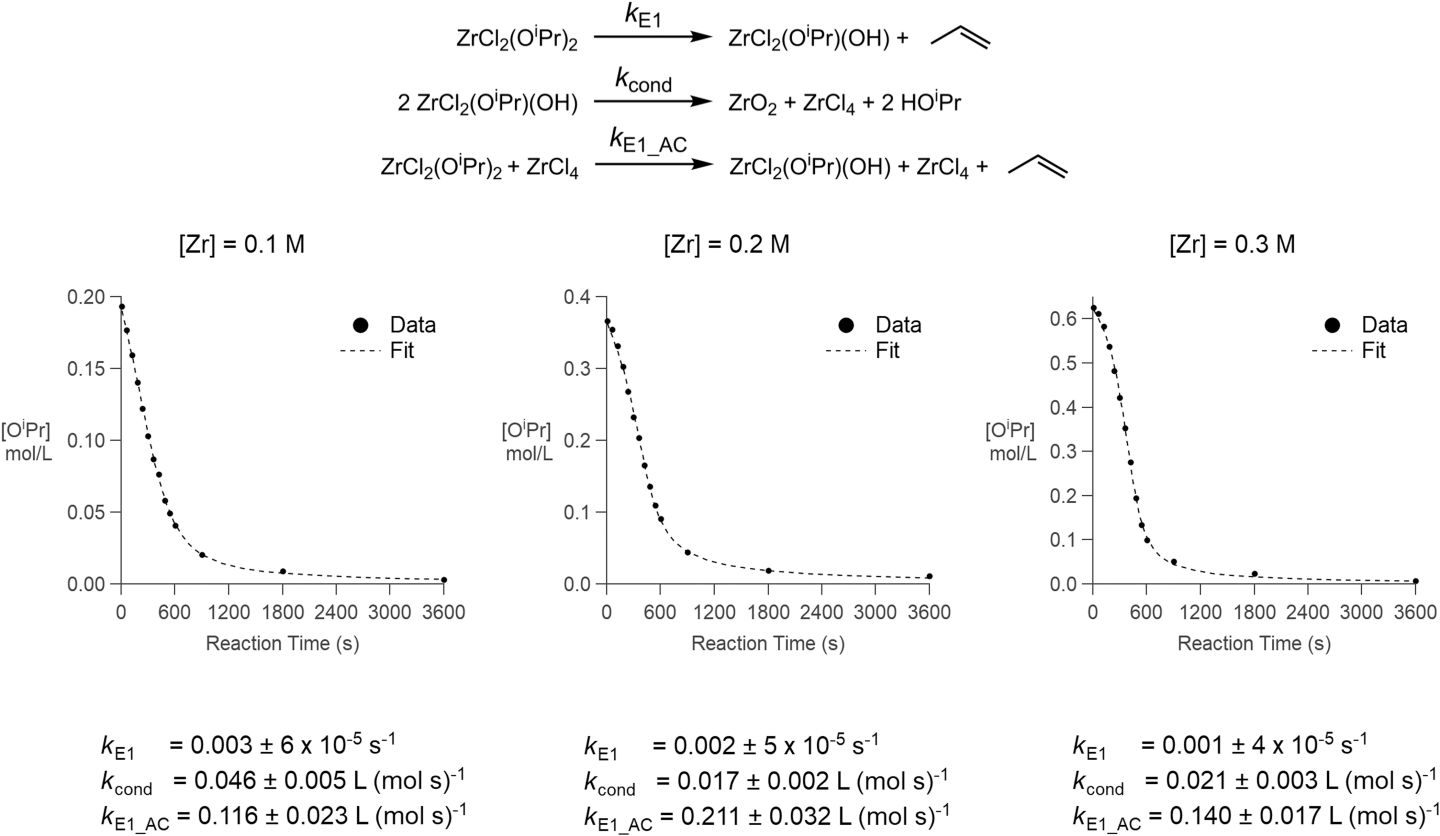
Fitted data for the decomposition of isopropoxide as a function of the reaction time for the three concentrations of zirconium in the presence of 11.4 equiv of TOPO represented in Figure S17. A set of reaction equations, including E1 elimination, condensation, and the E1 elimination auto-catalyzed by ZrCl_4_, was used as a model.

From the fitted rate constants, we conclude that the condensation step is fast with respect to the uncatalyzed E1 elimination. The auto-catalyzed E1 elimination is two orders of magnitude faster than the uncatalyzed first step. As the catalytic product, we hypothesize here ZrCl_4_ instead of ZrO_2_ or isopropanol. Indeed, during the decomposition of ZrCl_2_(O*^i^* Pr)_2_, the ^31^P NMR spectrum shows evidence of a transient ZrCl_3_(O*^i^* Pr) species.^21^ The third (auto-catalyzed) step can thus be conceived as the decomposition of ZrCl_3_(O*^i^* Pr) since

$${\mathrm{ZrCl}}_{2}{\left(O^{i}\mathrm{Pr}\right)}_{2}+{\mathrm{ZrCl}}_{4}\to 2{\mathrm{ZrCl}}_{3}\left(O^{i}\mathrm{Pr}\right)$$ 9

As we have observed above, ZrCl_3_(O*^i^* Pr) has faster decomposition kinetics (*k*_1_) than ZrCl_2_(O*^i^* Pr)_2_. The proposed mechanism is thus consistent with all experimental observations and is further supported by the consistency in the fitted rate constants.

Hafnium exhibits the same intermediates and decomposition products as zirconium.^21^ Its decomposition kinetics, shown in Figure 1, can be fitted using the FW two-step model, similar to zirconium, whereas this was not the case for titanium. Therefore, we infer that the formation of hafnia follows the same steps as elucidated for zirconium but with slightly slower kinetics.

After completion of the reaction, the nanocrystals were isolated and purified by precipitation with acetone, followed by several cycles of redispersion in toluene and precipitation with acetone. The nanocrystals were characterized by powder X-ray Diffraction (PXRD) and Transmission Electron Microscopy (TEM). For two equivalents of TOPO and down to 225 °C, the anatase crystal phase is still detected, while no particles were obtained at 200 °C after 3 h (Figure S21). The shape of the nanocrystals in TEM was irregular, as expected from previous literature (Figure S23). We estimated the size of the crystals by using the Scherrer equation on the anatase reflection at 25° (Figure S22). An increasing amount of TOPO leads to a decrease in the nanocrystal size, with the effect being more pronounced at a lower temperature. It is conceivable that the adsorption–desorption kinetics of the TOPO ligand to the nanocrystal surface slows at lower temperatures, therefore impeding growth. For zirconia, the tetragonal crystal phase is consistently retrieved (Figure S25). For the reactions with octadecane as a co-solvent, the nanocrystals are less colloidally stable, and we find that the injection procedure also negatively impacts the quality of the nanocrystals (Figure S26). Hence, the procedure outlined here was necessary to obtain accurate kinetics but is less suitable for nanocrystal production. For the syntheses in pure TOPO using different metal concentrations, the size distribution analysis of TEM images suggests that the size of the nanocrystals increases with the metal concentration for both Ti and Zr (Figures S24 and S28).

So far, we discussed the data obtained from commercially available isopropoxides. We also synthesized Zr­(O*^i^* Pr)_4_·*^i^* PrOH and Hf­(O*^i^* Pr)_4_·*^i^* PrOH according to established procedures (Figures S18–S19)^49^ and observed much slower decomposition kinetics (up to 6 times slower). Nevertheless, the synthesized precursors gave the same trends as the commercial ones: hafnium isopropoxide decomposes slower than zirconium isopropoxide (Figure S20). These results explain certain observations during the synthesis of core/shell nanocrystals. We observed secondary nucleation of the shelling material when we used commercial Hf­(O*^i^* Pr)_4_·*^i^* PrOH but not when using the in-house synthesized one.^30^ The faster precursor decomposition of commercial Hf­(O*^i^* Pr)_4_·*^i^* PrOH leads to a buildup of decomposed precursor, which is not consumed fast enough by growth on existing cores and instead nucleates new particles. Similar phenomena have been described in the literature.^50–54^ The same reasoning explains why we observed secondary nucleation with zirconia as shelling material but not with hafnia (the latter having slower kinetics).

## Conclusions

We quantified the decomposition kinetics of group 4 metal isopropoxides by monitoring the reaction via ^1^H NMR. Under identical conditions, titanium reacts an order of magnitude faster than zirconium while hafnium is slightly slower than zirconium. In the case of titania, the reaction follows an S_*N*_1 mechanism, and the addition of TOPO accelerates the reaction by stabilizing the positive charge in the transition state. When the ratio of chloride to isopropoxide was varied in the precursor series TiCl_3_(O*^i^* Pr), TiCl_2_(O*^i^* Pr)_2_, and TiCl­(O*^i^* Pr)_3_, the initial rate decreased with decreasing chloride content since the electron-withdrawing chloride makes the titanium metal more Lewis acidic, thus stabilizing the negative charge in the transition state. Zirconium follows a more complicated mechanism involving an E1 elimination, a condensation, and an auto-catalysis step. While the above insights remain true for the transition state of the E1 elimination of zirconium isopropoxide, the overall kinetics is mostly influenced by an auto-catalysis step, which appears to be inhibited by addition of TOPO. This can be accounted for by an additional step where the product capable of auto-catalysis is involved in an association equilibrium with TOPO. Overall, we provided a quantitative picture of the first step in metal oxide formation from metal isopropoxides. This opens the door toward a more rational design of complex architectures based on group 4 metal oxides such as ZrO_2_/HfO_2_ core/shell nanocrystals or ZrTiO_4_ nanocrystals.

## Experimental Section

### Materials

TiCl_4_ (Acros, 99.99%), Ti­(O*^i^* Pr)_4_ (Strem Chemicals, 98%), ZrCl_4_ (Strem Chemicals, 99.9%), and HfCl_4_ (Strem Chemicals, 99.9%) were used without further purification. Zr­(O*^i^* Pr)_4_·*^i^* PrOH (99.9%) and Hf­(O*^i^* Pr)_4_·*^i^* PrOH (99.9%) were both purchased from Sigma-Aldrich and Strem Chemicals, respectively, or synthesized following the procedure reported by Bradley et al.^55^ and Dhaene et al.^49^ Tri-*n*-octylphosphine oxide (Strem Chemicals, 99%) was recrystallized according to the procedure described by Owen et al.^56^ NMR measurements were performed in chloroform-*d* (Apollo Scientific, 99.8% and Euroisotop, 99.5 atom %) and benzene-*d*_6_ (Apollo Scientific, 99.5 atom %) that were dried using activated 4 Å molecular sieves to remove residual water and stored in the glovebox. Octadecane (Sigma-Aldrich, 99%), toluene (VWR chemicals, for HPLC 100%), acetone (Biosolve Chemicals), and cyclohexane (Honeywell, ≥99.7%) were used as received.

### General Instrumentation

#### Nuclear Magnetic Resonance (NMR)

Spectra were recorded at 298.15 K on a Bruker UltraShield 500 spectrometer operating at a ^1^H frequency of 500.13 MHz. ^1^H NMR spectra were acquired using standard 30 degree flip angle pulse sequences from the Bruker library with a D1 of 10 s for ^1^H NMR (zg30, 16 scans). Chemical shifts (δ) are given in parts per million (ppm), and the residual solvent peak was used as an internal standard (C_6_D_6_: δH = 7.16 ppm, CDCl_3_: δH = 7.26 ppm).

#### Powder X-ray Diffraction (PXRD)

Patterns were collected at room temperature in the transmission mode using a Stoe Stadi P diffractometer with a microfocused Cu–Kα-source (λ = 1.542) equipped with a DECTRIS MYTHEN 1K detector. The size of the nanoparticles was calculated using the Scherrer equation

10 $$d=\frac{0.9\lambda }{\beta \cos\left(\theta \right)}$$

where λ is the X-ray wavelength in Å, θ is the diffraction angle, and β is the full width at half-maximum (FWHM) in radians, calculated from the width of the Gaussian fit through

11 $$f\left(x\right)=y_{0}+Ae^{-\left({\left(x-x_{0}\right)}^{2}/width^{2}\right)},\mathrm{and},\beta =2width\sqrt{\ln2}$$

#### Bright Field-Transmission Electron Microscopy (BF TEM)

Imaging was carried out in JEOL JEM-F200 operated in the TEM mode at a beam energy of 200 kV. The nanoparticles were dissolved in cyclohexane to obtain solutions with concentrations between 0.25 and 1 mg/mL. Two drops of the solution were deposited on a carbon grid Quantifoil R 1.2/1.3 Cu 200 + 2 nm C.

#### Gas Chromatography (GC-FID)

GC-FID was measured on a gas chromatograph (SRI 8610C, SRI instruments) equipped with a Haysep D column (3 m 2 mm ID Mesh 80/100) and an FID detector. N_2_ was used as carrier gas with a wt rate of 1 mL/min. From the gas phase in the reaction flask, samples (50 μL) were taken with a gastight syringe and injected into headspace crimp vials (10 mL) filled with nitrogen. From the vial, 1 mL was injected with an autosampler (HT2000H, HTA Instruments). The separation of the products was achieved with a temperate gradient starting from 70 (held for 2 min) and then heating to 270 °C at a rate of 10 °C min^–1^. Commercial isopropyl chloride (99%, Sigma-Aldrich) and propene (Pangas) were used as references.

### Nanocrystal Synthesis

A detailed description is provided in the Supporting Information (SI). Briefly, in a 25 mL 3-neck round bottom flask, liquid octadecane and recrystallized TOPO were added with the metal chloride precursor. In a 20 mL vial, M­(O*^i^* Pr)_4_ and TOPO were mixed together, corresponding to 150% of the needed amount for hot injection into the reaction mixture. Once the reaction mixture reached the desired temperature, the hot M­(O*^i^* Pr)_4_-TOPO solution was injected into the flask, which was the start of the reaction (*t* = 0 s). During the reaction, small aliquots were extracted and analyzed by ^1^H NMR spectroscopy. The reaction was stirred for 3 h. After the reaction time had elapsed, the reaction was allowed to cool to around 100 °C, and 1.5 mL of toluene was injected into the reaction mixture. The nanocrystals were purified following the procedure reported by De Keukeleere et al.,^18^ before suspending them in cyclohexane (5 mL).

### Kinetics Data Collection

Small aliquots (ca. 0.1 mL) were extracted from standard reactions at predetermined time points, transferred to an air-free septum-sealed NMR tube containing deuterated chloroform or deuterated benzene (0.5 mL), and analyzed by ^1^H NMR spectroscopy. ^1^H NMR spectra were collected using 16 scans and a delay time (D1) of 10 s, which was determined to be ideal for quantitative measurements by acquiring spectra with different D1 values until the integration no longer changed. All spectra were phase and background corrected before integration of the ^1^H peaks indicative of MCl_4–*x*_(O*^i^* Pr)_*x*_. These integrations were compared against the CH_3_ signal of TOPO and octadecane (0.8–0.95 ppm), whose concentrations remain constant throughout the reaction and appear at the same chemical shift. In the case of zirconium, the H of the CH group in the isopropoxide appears between 4.25 and 4.70 ppm in CDCl_3_, while in the case of hafnium, it appears between 4.35 and 4.80 ppm. For titanium, the determination of the isopropoxide integral was more complicated. This resonance is very close to the protons of the CH_2_ group of propene (4.90–5.07 ppm), which form during the reaction (Figure S1). The contribution of propene is subtracted from the broad TiCl_4–*x*_(O*^i^* Pr)_*x*_ resonance between 4.35 and 5.7 ppm by using the integral of the CH group of propene, appearing at 5.75–5.9 ppm.

### Kinetic Analysis with COPASI

The open-source software COPASI^57^ was used to fit the kinetics data. Pseudo-elementary step mechanisms were conceived for the precursor decomposition reaction, inputted into the software, and tested against the kinetics data. Data were inputted as concentration (mol/L) versus time (second). Mass balance was used for parameter estimation in the time-dependent fittings, employing the particle swarm method with an iteration limit of 2000 and a swarm size of 50. Rate constants were collected from the fits and used to quantitatively compare the effects of different experimental variables on the reaction kinetics.

## Supplementary Material





## Notes

^1^H NMR spectra of the aliquots and precursor decomposition curves; additional fittings and characterization of the final nanoparticles with Bright field TEM and powder XRD (PDF)
Input (.txt) and COPASI (.cps) files together with a Readme (ZIP)
