# Supplementary material for: From Kinetics to Molecular-Level Insights into Group 4 Metal Oxide Nanocrystal Synthesis
Source: ACS Mater Au. 2025 May 29;5(4):709–17. doi: 10.1021/acsmaterialsau.5c00032 (PMC12257421; doi:10.1021/acsmaterialsau.5c00032)
Supplement: Supplementary file 1 [file mg5c00032_si_001.pdf]

Supporting Information: From Kinetics to Molecular-Level Insights into Group 4 Metal Oxide Nanocrystal Synthesis

Carlotta Seno,† Christopher B Whitehead,†,‡ David E Salazar Marcano,† Ian Chaon,† and Jonathan De Roo*,†

†*Department of Chemistry, University of Basel, Mattenstrasse 22, 4058 Basel, Switzerland*

‡*Department of Chemistry, Union College, Schenectady, NY 12305, United States*

E-mail: Jonathan.DeRoo@unibas.ch

Contents

1 Nanocrystal Synthesis Procedure **S-3**

1.1 Synthesis with octadecane as co-solvent S-3

1.2 Synthesis in pure TOPO S-4

2 From raw NMR data to kinetics plots **S-6**

3 Influence of temperature **S-9**

4 Titania **S-10**

5 Zirconia **S-13**

6 Synthesized isopropoxides **S-15**

7 Nanocrystal characterization **S-17**

7.1 Titania S-17

7.2 Zirconia S-19

References **S-20**

1 Nanocrystal Synthesis Procedure

1.1 Synthesis with octadecane as co-solvent

In a 25-mL 3-neck round bottom flask, liquid octadecane (heated to 70 °C over 20 min) and recrystallized TOPO were mixed according to the amounts in Table S1, while varying the amount of TOPO the volume of octadecane was adjusted to keep the total volume of the reaction always constant. The metal chloride was added together with a glass-coated stirring bar (Table S2). The three-neck flask was sealed with a rubber septum, a condenser, and a thermowell. The condenser and thermowell were connected using high-temperature vacuum grease. Next, in a 20-mL vial, the $M(O^{i}Pr)_4$ and TOPO were mixed together, corresponding to 150% of the needed amount for hot injection into the reaction mixture (Table S2). A stirring bar was added, and the vial was sealed with a septum. The three-neck set-up and the vial were taken out from the glovebox. The three-neck set-up was connected to the Schlenk line, filled with Argon, and heated with a heating mantle controlled by a thermocontroller to the desired temperature. The metal isopropoxide in TOPO solution was stirred and heated to 100 °C in an aluminum block for 20 minutes. Once the reaction mixture reached the desired temperature, the $M(O^{i}Pr)_4$-TOPO solution was injected into the flask (Table S2), making sure the tip of the syringe was in the reaction mixture, to avoid the evaporation of the oxide precursor. The start of the reaction time was set to immediately after the injection. During the reaction, small aliquots (ca. 0.1 mL) were extracted and analyzed by ^{1}H NMR spectroscopy. The reaction was then stirred for 3 hours. After the reaction time had elapsed, the reaction was allowed to cool down to around 100 °C and 1.5 mL of toluene was injected into the reaction mixture to prevent the solidification of TOPO. The nanocrystals were purified following the procedure reported by De Keukeleere *et al.*[S1] Acetone was added to the reaction mixture, in a volume ratio 2:1, giving a precipitate after centrifugation (8000 rcf, 3 min). The solid was then suspended back in toluene (1.5 mL), sonicated for 10 minutes, and precipitated again with acetone. This step was repeated once more, before suspending

the nanoparticles in cyclohexane (5 mL).

Table S1: Amounts of TOPO and octadecane (OD) added to the three-neck flask. In this work, the equivalents of TOPO were chosen to be 2, 4, or 6, consequently, to have a constant total reaction volume of 10 mL, 12, 9.3, or 6.6 were the equivalents of octadecane. Note that for the reactions with 1 :1 ratio of MCl_4 : $M(O^iPr)_4$, one equivalent of TOPO (0.77 g, 2 mmol) was added to the flask during the injection together with the $M(O^iPr)_4$ precursor, the remaining equivalents of TOPO were added to the flask together with OD.

TOPO (equiv.)	TOPO (mmol)	TOPO (g)	OD (equiv.)	OD (mmol)	OD (ml)
2	4	1.55	12	24	7.86
4	8	3.09	9.3	18.6	6.09
6	12	4.64	6.6	13.2	4.32

Table S2: Amounts of MCl_4 precursor added to the round bottom flask, of $M(O^iPr)_4$ (150% of the needed amount for the hot injection) and TOPO (150% of the needed amount for the hot injection) added to the vial. In the last column, the Volume of the $M(O^iPr)_4$-TOPO solution injected into the reaction mixture. In all cases, the total amount of TOPO is of 2 equivalents with respect to the total metal content.

	Cl:OR	MCl_4 flask	$M(O^iPr)_4$ vial (150%)	TOPO vial (150%)	Injection
Ti	3:1	0.165 mL (1.5 mmol)	0.222 mL (0.75 mmol)	0.580 g (1.5 mmol)	0.6 mL
	1:1	0.110 mL (1 mmol)	0.444 mL (1.5 mmol)	1.160 g (3 mmol)	1.20 mL
	1:3	0.055 mL (0.5 mmol)	0.666 mL (2.25 mmol)	1.739 g (4.5 mmol)	1.80 mL
Zr	3:1	0.350 g (1.5 mmol)	0.291 g (0.75 mmol)	0.580 g (1.5 mmol)	0.65 mL
	1:1	0.233 g (1 mmol)	0.582 g (1.5 mmol)	1.160 g (3 mmol)	1.30 mL
	1:3	0.117 g (0.5 mmol)	0.872 g (2.25 mmol)	1.739 g (4.5 mmol)	1.95 mL
Hf	1:1	0.320 g (1 mmol)	0.712 g (1.5 mmol)	1.160 g (3 mmol)	1.35 mL

1.2 Synthesis in pure TOPO

The reactions were conducted in pure TOPO at 340 °C for Zr and at 250 °C for Ti. The same setup and procedure as the ones described for the syntheses with octadecane as co-solvent were used, but without the addition of octadecane. The mol amounts of MCl_4 and $M(O^iPr)_4$

were adjusted to vary the metal concentration according to Table S3. To calculate the metal concentration in mol/L, the density of TOPO has been approximated to its solid state value (0.88 g cm^{-3}). After reaching the reaction temperature, the $M(O^iPr)_4$-TOPO solution was injected and the reaction was run for 3 hours. The nanocrystals were purified in the same way as described previously for the synthesis with octadecane as co-solvent.

Table S3: Amounts of MCl_4, $M(O^iPr)_4$ (150% of the needed amount), TOPO added to the flask and TOPO added to the vial (2 equivalents with respect to the amount of $M(O^iPr)_4$; 150% of the needed amount) used to vary the metal concentration in the reaction mixture.

	[M] (mol/L)	MCl_4	$M(O^iPr)_4$ (150%)	TOPO flask	TOPO vial (150%)	Injection
	0.1	0.055 mL (0.5 mmol)	0.222 mL (0.75 mmol)	8.2 g (21.3 mmol)	0.580 g (1.5 mmol)	0.6 mL
Ti	0.2	0.110 mL (1 mmol)	0.444 mL (1.5 mmol)	8.0 g (20.8 mmol)	1.16 g (3 mmol)	1.2 mL
	0.3	0.220 mL (2 mmol)	0.888 mL (3 mmol)	8.5 g (21.9 mmol)	2.3 g (6 mmol)	2.4 mL
	0.1	0.117 g (0.5 mmol)	0.291 g (0.75 mmol)	8.4 g (21.3 mmol)	0.580 g (1.5 mmol)	0.65 mL
Zr	0.2	0.233 g (1 mmol)	0.582 g (1.5 mmol)	8.0 g (20.8 mmol)	1.16 g (3 mmol)	1.3 mL
	0.3	0.466 g (2 mmol)	1.16 g (3 mmol)	8.5 g (21.9 mmol)	2.3 g (6 mmol)	2.6 mL

2 From raw NMR data to kinetics plots

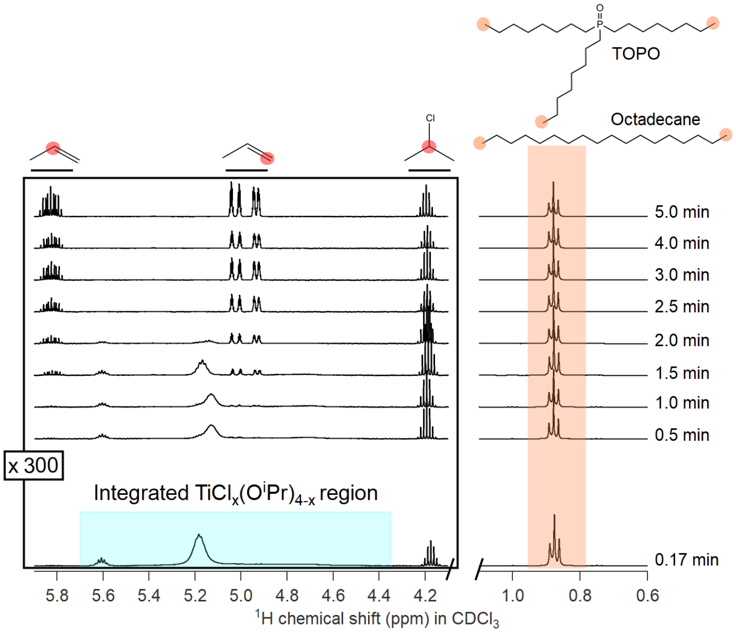

Figure S1: ^{1}H NMR of the aliquots collected during the synthesis of TiO_2 at 275 °C with 2 eq. of TOPO. The peak of TOPO and octadecane is used as internal standard to calibrate the integrals. The isopropoxide groups of titanium chloroalkoxide species appear between 4.35-5.7 ppm, the terminal alkene of propene (at 5 ppm) is subtracted by internal calibration with the peak at 5.8 ppm. The decomposition of the isopropoxide groups is shown in Figure 1.

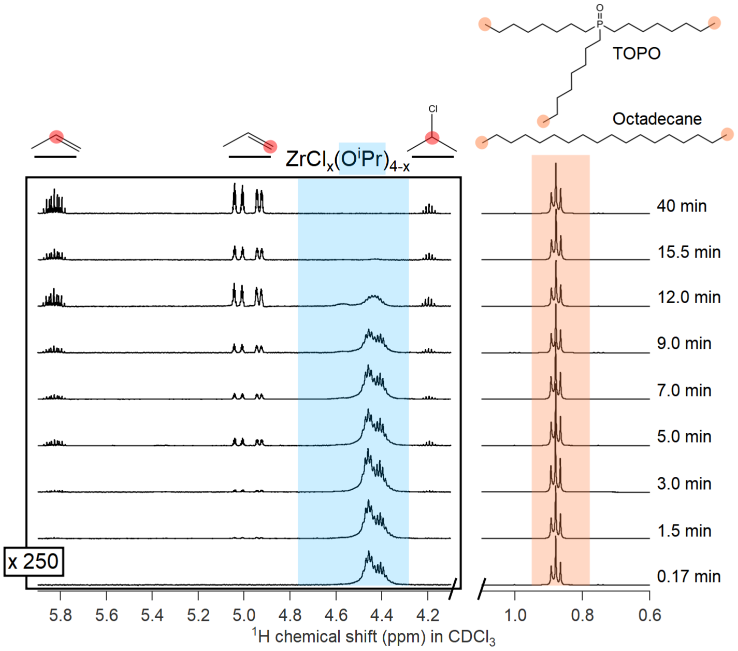

Figure S2: ^{1}H NMR of the aliquots collected during the synthesis of ZrO_2 at 275 °C with 2 eq. of TOPO. The peak of TOPO and octadecane is used as internal standard to calibrate the integrals. The decomposition of the isopropoxide groups is shown in Figure 1.

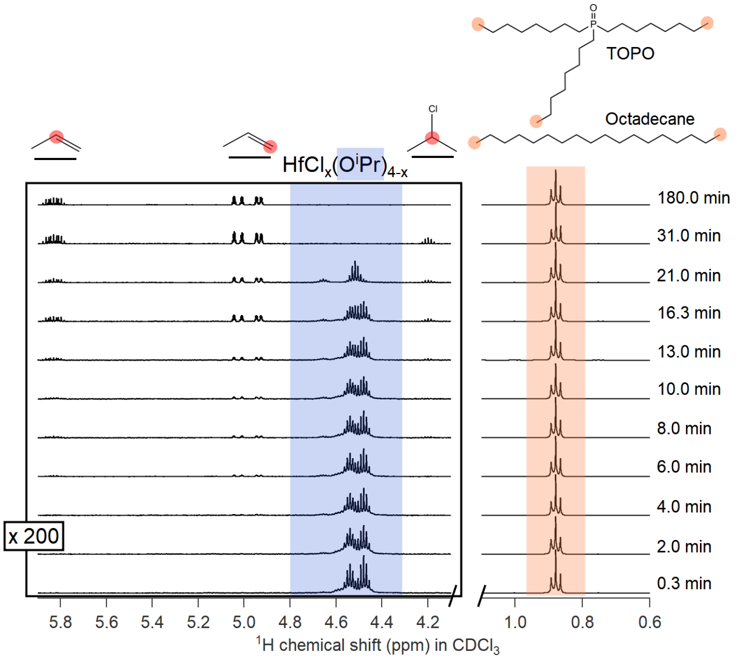

Figure S3: ^{1}H NMR of the aliquots collected during the synthesis of HfO_2 at 275 °C with 2 eq. of TOPO. The peak of TOPO and octadecane is used as internal standard to calibrate the integrals. The decomposition of the isopropoxide groups is shown in Figure 1.

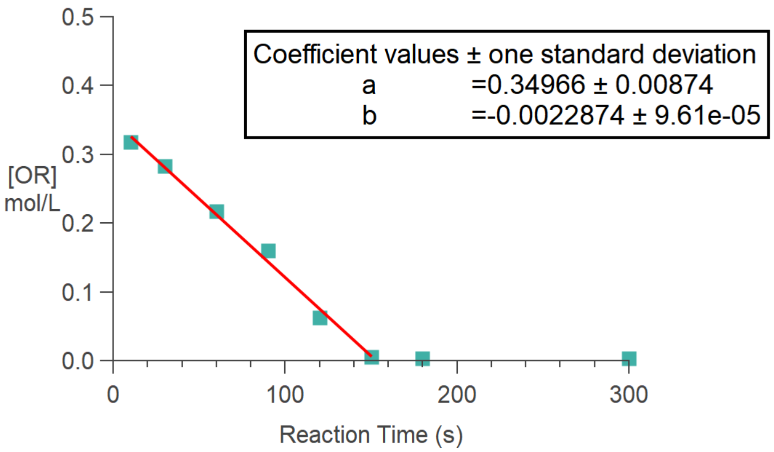

Figure S4: Precursor decomposition of titanium at 275 °C with 2 eq. of TOPO shown in Figure 1. The isopropoxide disappearance was fitted using a linear curve.

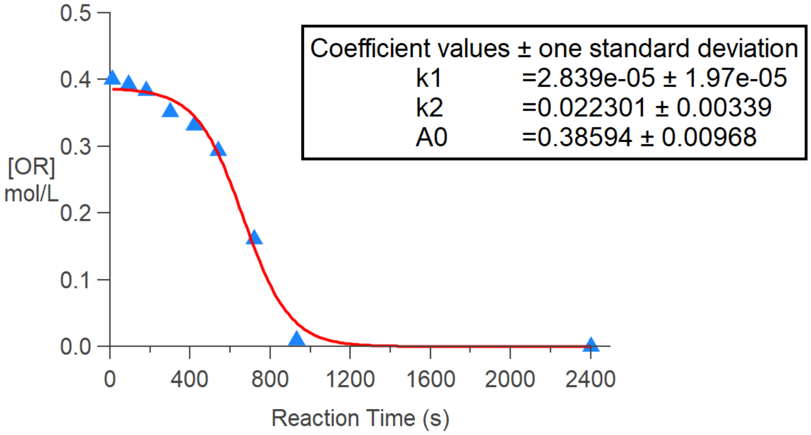

Figure S5: Precursor decomposition of zirconium at 275 °C with 2 eq. of TOPO shown in Figure 1. The isopropoxide disappearance was fitted using the Finke-Watzky two-step model.

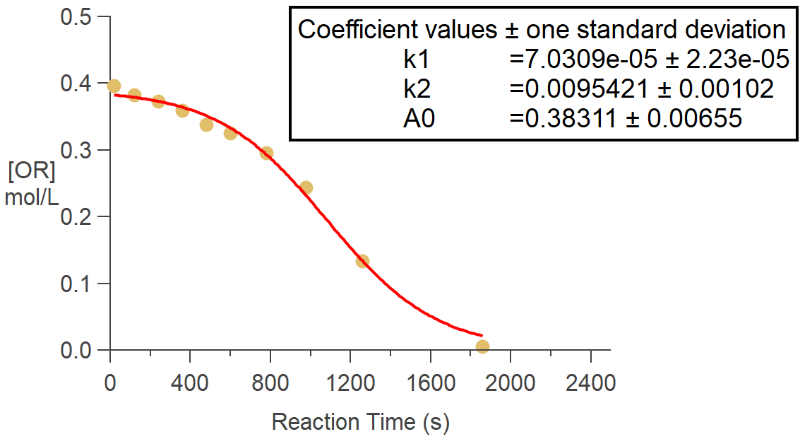

Figure S6: Precursor decomposition of hafnium at 275 °C with 2 eq. of TOPO shown in Figure 1. The isopropoxide disappearance was fitted using the Finke-Watzky two-step model.

3 Influence of temperature

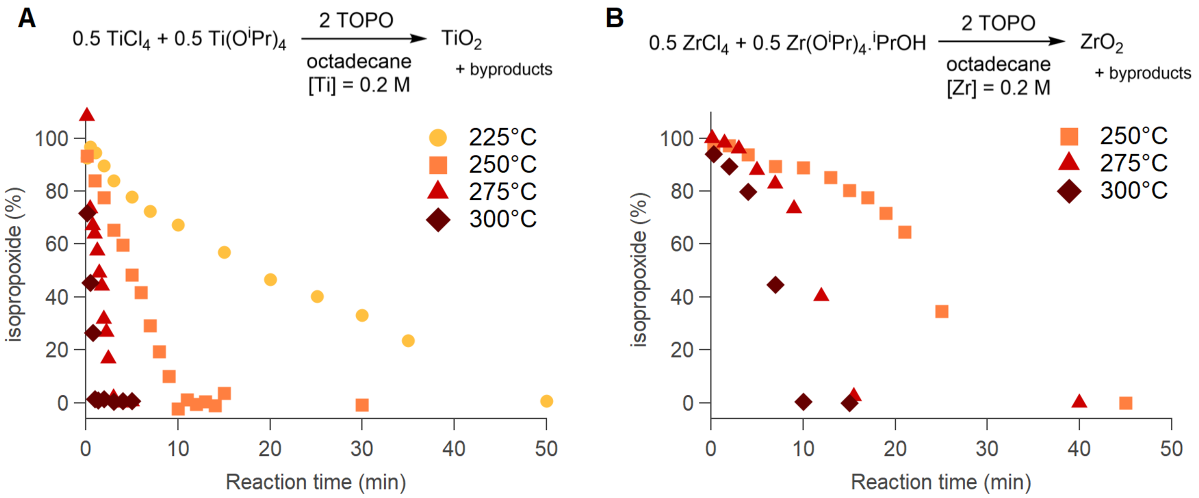

Figure S7: Precursor decomposition as a function of the reaction temperature for (A) titanium and (B) zirconium oxide.

4 Titania

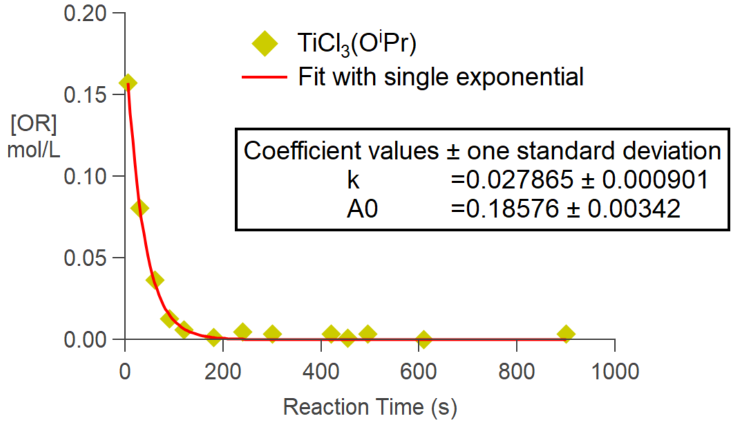

Figure S8: Precursor decomposition of titanium at 250 °C with 2 eq. of TOPO and starting from $TiCl_3(O^iPr)$ shown in Figure 2C. The isopropoxide disappearance was fitted using a single exponential function.

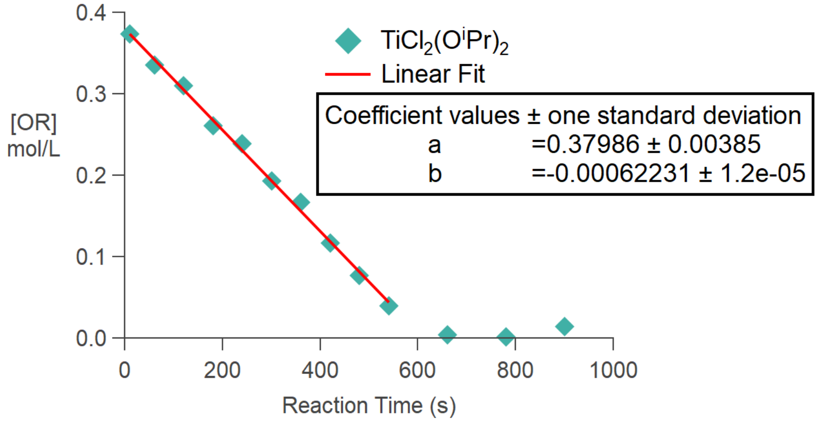

Figure S9: Precursor decomposition of titanium at 250 °C with 2 eq. of TOPO and starting from $TiCl_2(O^iPr)_2$ shown in Figure 2C. The isopropoxide disappearance was fitted using a linear curve.

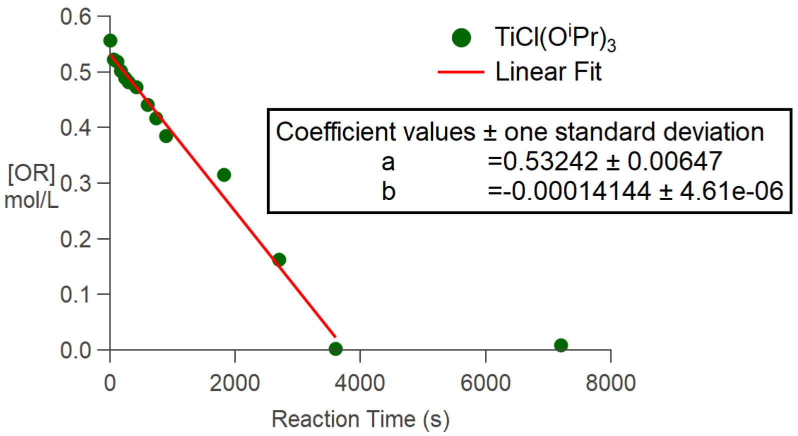

Figure S10: Precursor decomposition of titanium at 250 °C with 2 eq. of TOPO and starting from $TiCl(O^iPr)_3$ shown in Figure 2C. The isopropoxide disappearance was fitted using a linear curve.

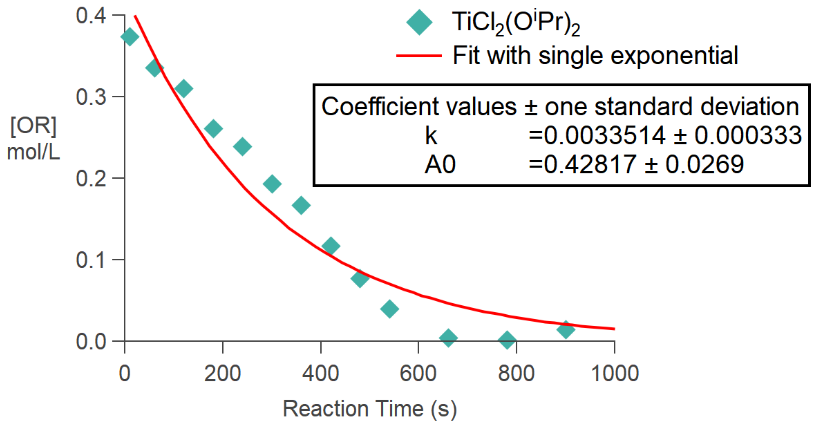

Figure S11: Precursor decomposition of titanium at 250 °C with 2 eq. of TOPO and starting from $TiCl_2(O^iPr)_2$ shown in Figure 2C. The isopropoxide disappearance was fitted using a single exponential function.

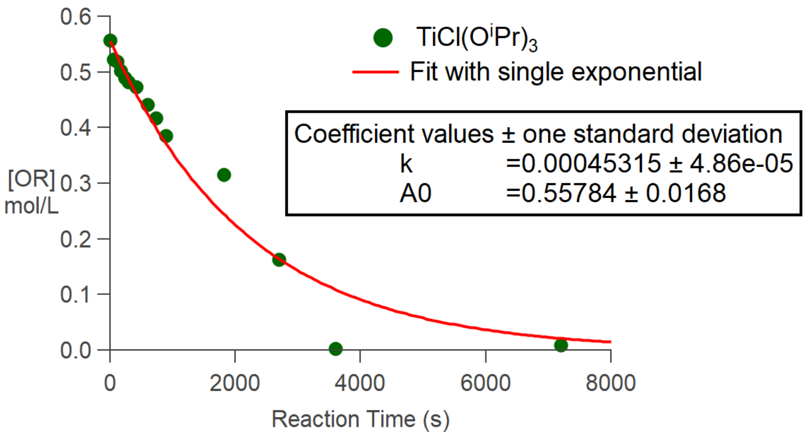

Figure S12: Precursor decomposition of titanium at 250 °C with 2 eq. of TOPO and starting from TiCl(O^{i}Pr)$_3$ shown in Figure 2C. The isopropoxide disappearance was fitted using a single exponential function.

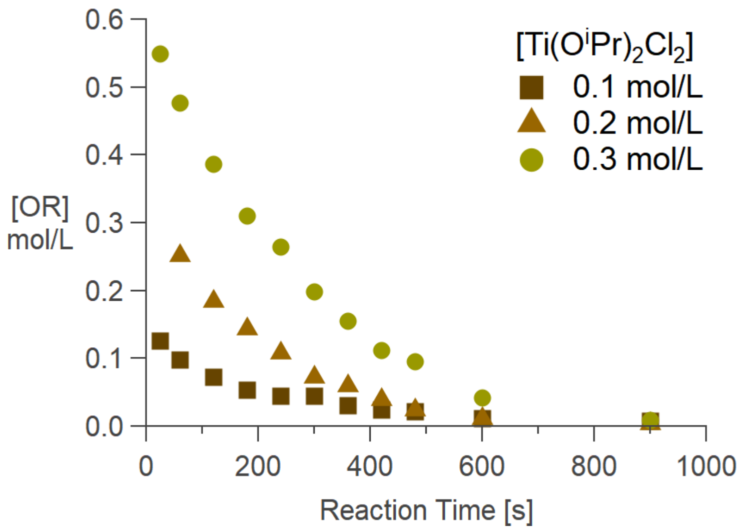

Figure S13: Precursor decomposition for titania varying the titanium concentration in the presence of 11.4 eq. of TOPO at 250 °C.

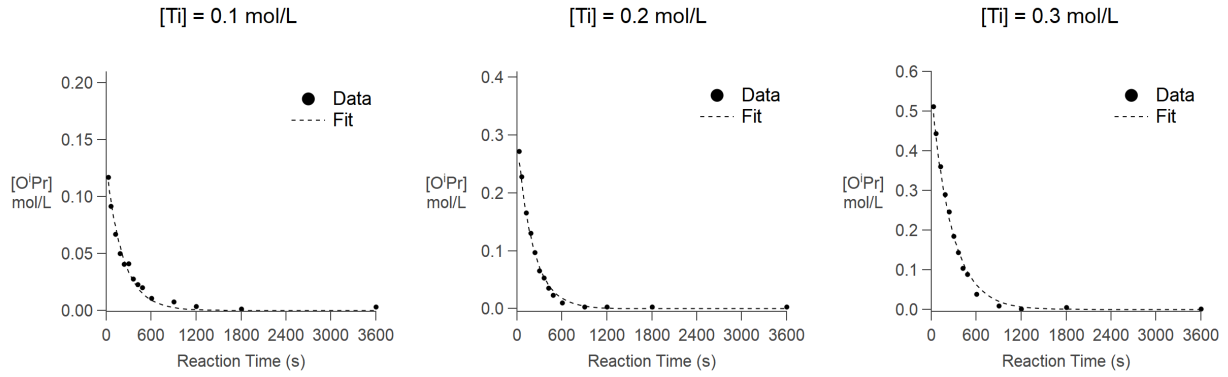

Figure S14: Fitted data for the decomposition of isopropoxide as a function of the reaction time for the three concentrations of titanium in the presence of 11.4 eq. of TOPO at 250 °C represented in Figure S13. A first order reaction (Equation (5)) was used as model, where $TiCl_2(O^iPr)_2$ forms TiO_2 and isopropyl chloride.

5 Zirconia

Table S4: Calculations of $\ln(k_1)$ and $\ln(k_2)$ from the values of the rate constants obtained by fitting the zirconium data in Figure S7B with FW two-step model. Plots of $\ln(k)$ vs 1/T are shown in Figures S15 and S16. From their slope is possible to calculate the activation energy (E_a) using the formula in Equation (1), derived from Arrhenius equation (Equation (2)), where R is the gas constant = 8.314 J(mol K)$^{-1}$, and A the Arrhenius factor.

T °C	T K	1/T K^{-1}	k_1 s^{-1}	$\ln(k_1)$ –	k_2 L (mol s)$^{-1}$	$\ln(k_2)$ –
250	523	0.00191	1.40 10^{-5}	-11.2	0.011	-4.51
275	548	0.00182	2.80 10^{-5}	-10.5	0.022	-3.82
300	573	0.00175	3.70 10^{-5}	-10.2	0.040	-3.22

$$slope = \frac{-E_a}{R} \qquad E_a = -slope \cdot R \tag{1}$$

$$k = A \cdot \exp\left(-\frac{E_a}{RT}\right) \qquad \ln(k) = -\frac{E_a}{R} \cdot \frac{1}{T} + \ln(A) \tag{2}$$

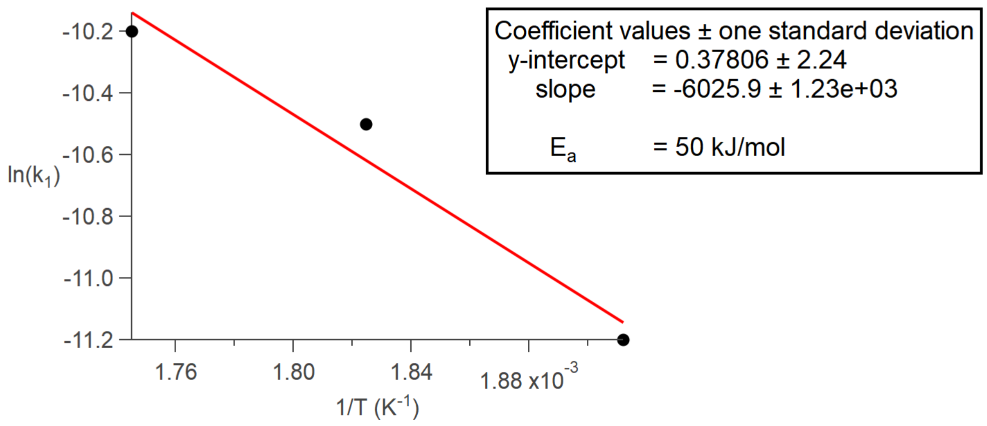

Figure S15: $\ln(k_1)$ plotted as a function of (1/T) as explained in Table S4.

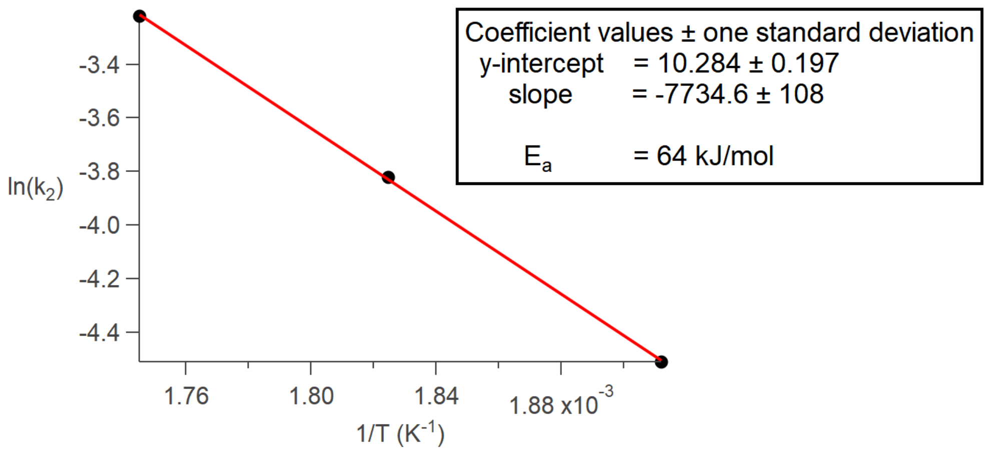

Figure S16: $\ln(k_2)$ plotted as a function of (1/T) as explained in Table S4.

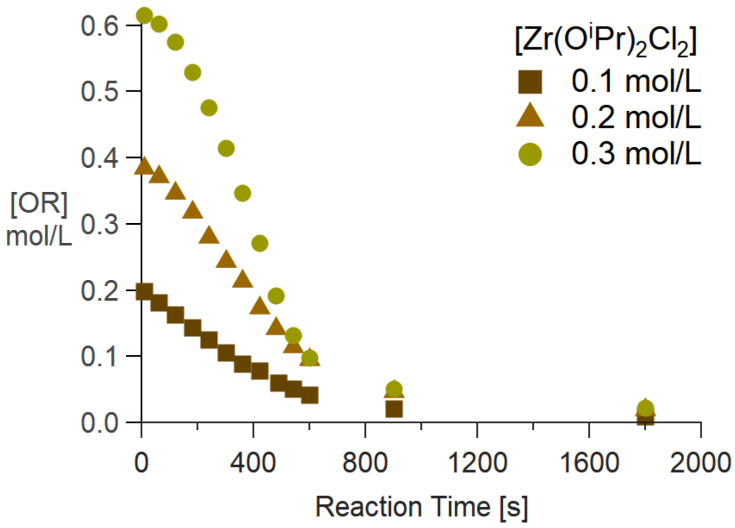

Figure S17: Precursor decomposition for zirconia varying the zirconium concentration in the presence of 11.4 eq. of TOPO at 340 °C.

6 Synthesized isopropoxides

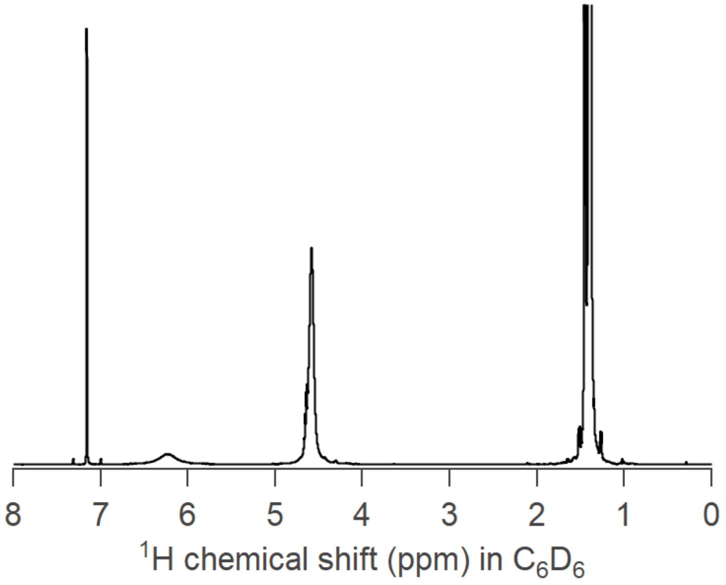

Figure S18: ^{1}H NMR of in-house synthesized $Zr(O^iPr)_4$.

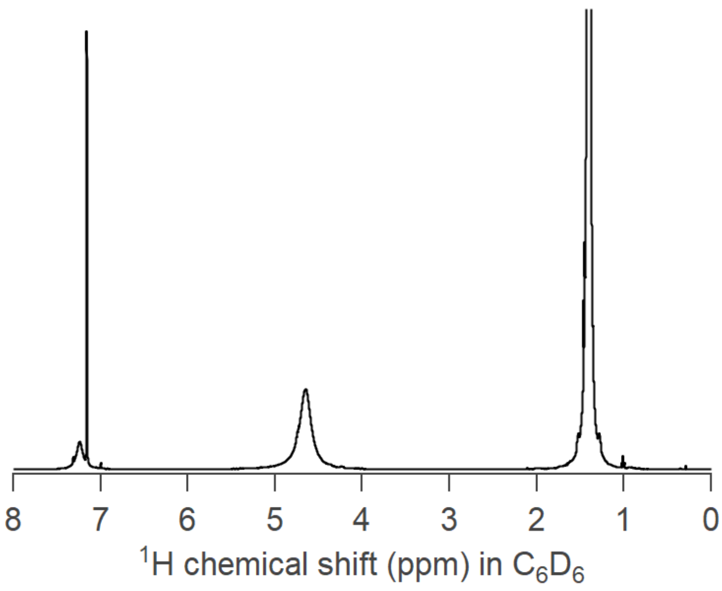

Figure S19: ^{1}H NMR of in-house synthesized $Hf(O^iPr)_4$.

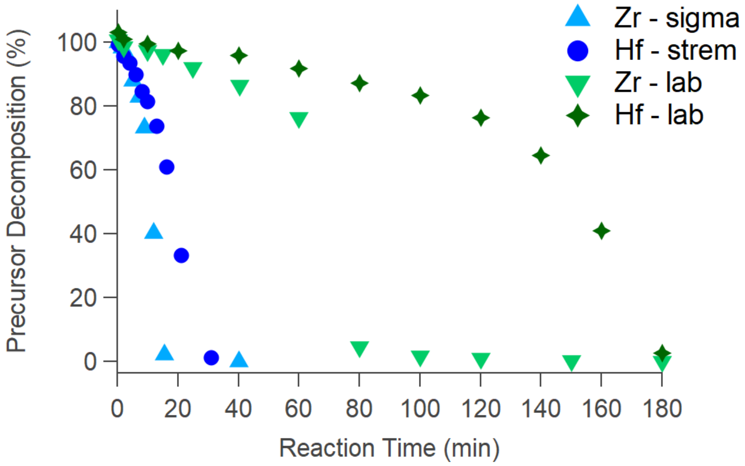

Figure S20: Precursor decomposition of zirconium and hafnium monitored *via* ^{1}H NMR over the reaction time, with the syntheses conducted at 275 °C with 2 eq. of TOPO and with a metal concentration equal to 0.2 mol/L using commercial zirconium and hafnium isopropoxide precursors as well as in-house synthesized precursors (lab) following the procedure reported by Bradley *et al.*[S2] and adapted by Dhaene *et al.*[S3]

7 Nanocrystal characterization

7.1 Titania

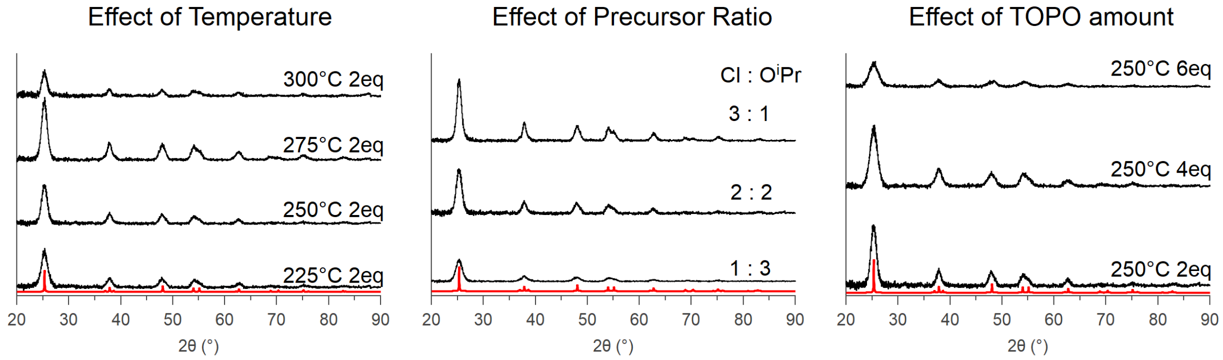

Figure S21: Powder XRD patterns of TiO_2 nanoparticles at different reaction conditions, together with the TiO_2 anatase reference in red.[S4] Under all conditions, the anatase crystal phase is retrieved.

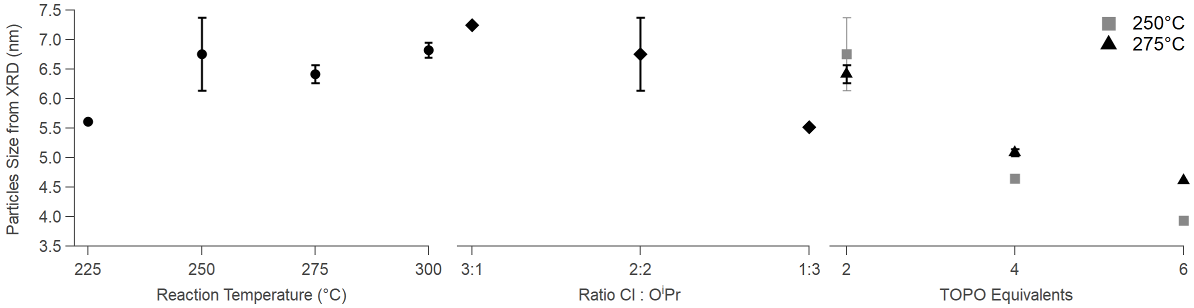

Figure S22: Dependence of TiO_2 crystal size calculated from the XRD patterns in Figure S21 when different reaction conditions were investigated: changing reaction temperature with 2 eq. of TOPO, the ratio between $TiCl_4$ and $Ti(O^iPr)_4$ at 250 °C, and different TOPO equivalents at 250 °C and 275 °C.

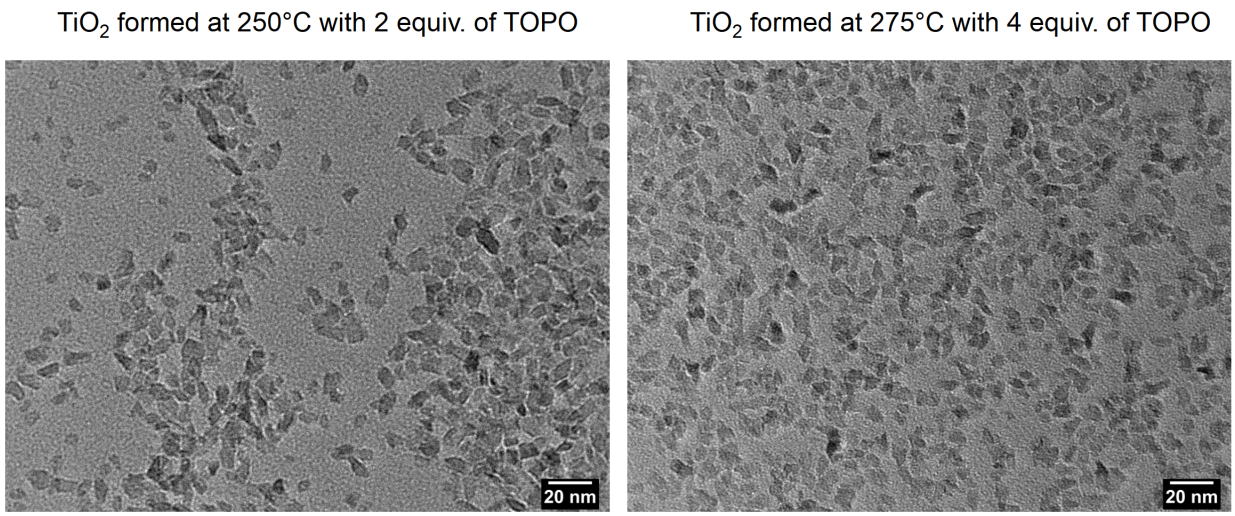

Figure S23: Bright-Field TEM images of TiO_2 nanoparticles formed at different reaction conditions.

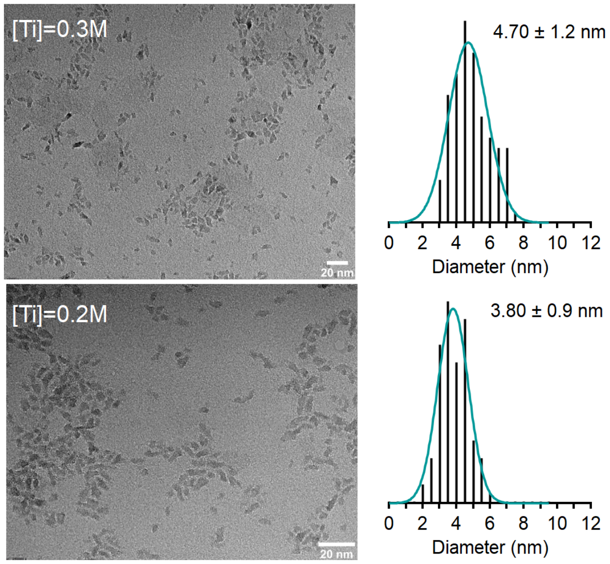

Figure S24: Size distribution of TiO_2 nanocrystals obtained from pure TOPO at 250 °C with different metal concentrations. The histograms are based on more than 80 nanoparticles in bright-field TEM images.

7.2 Zirconia

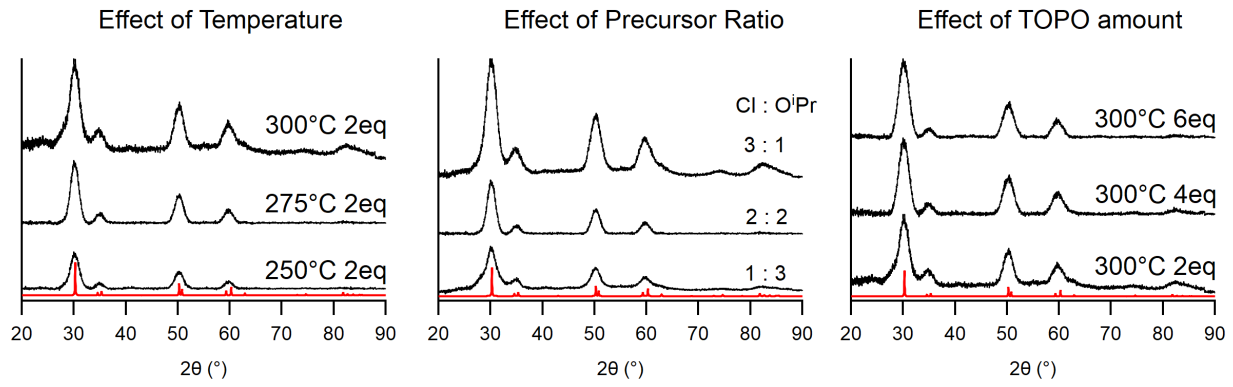

Figure S25: Powder XRD patterns of ZrO_2 nanoparticles at different reaction conditions, together with the ZrO_2 tetragonal reference in red.[S5] The tetragonal crystal phase is present under all conditions.

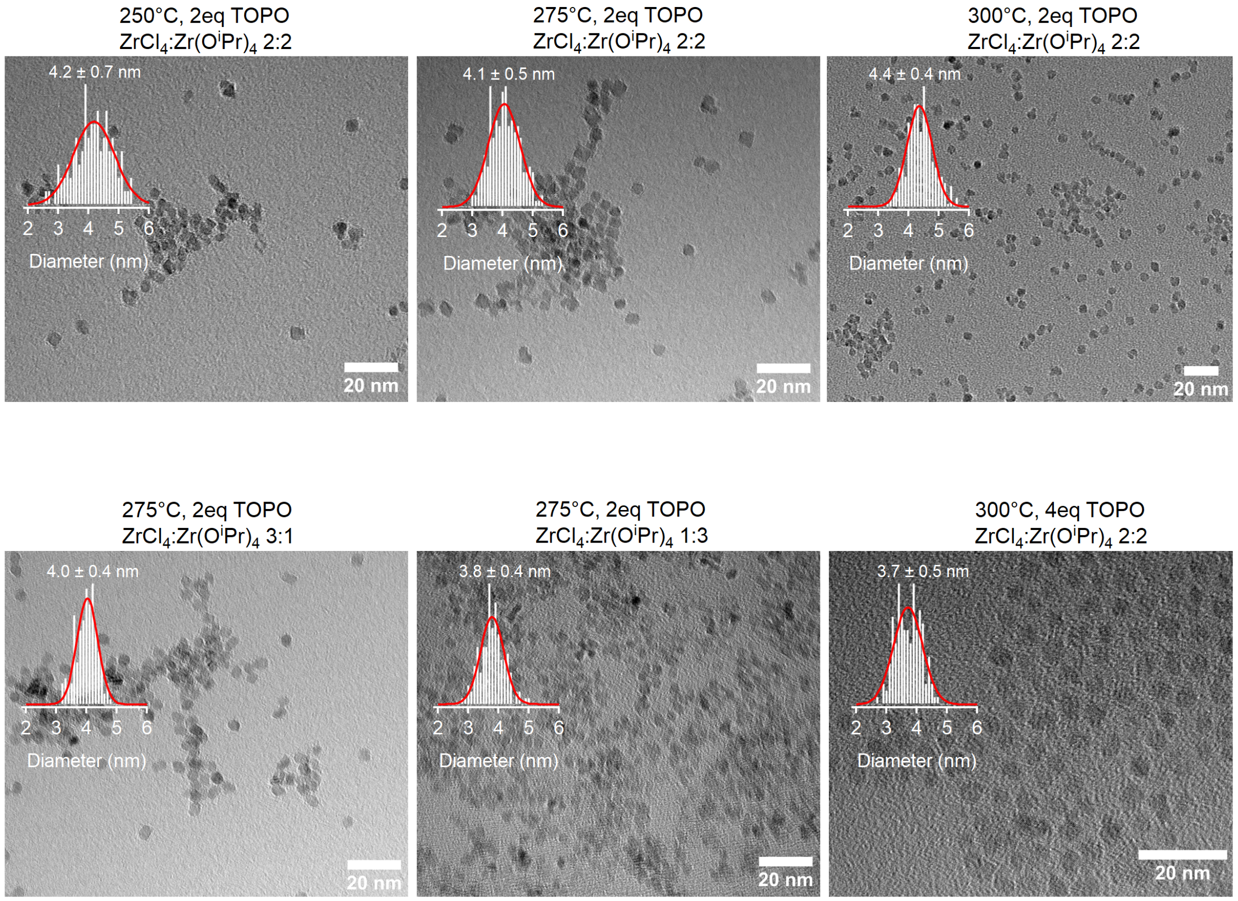

Figure S26: Bright-Field TEM images of ZrO_2 nanoparticles synthesized at different reaction conditions. The histograms are based on more than 100 particles.

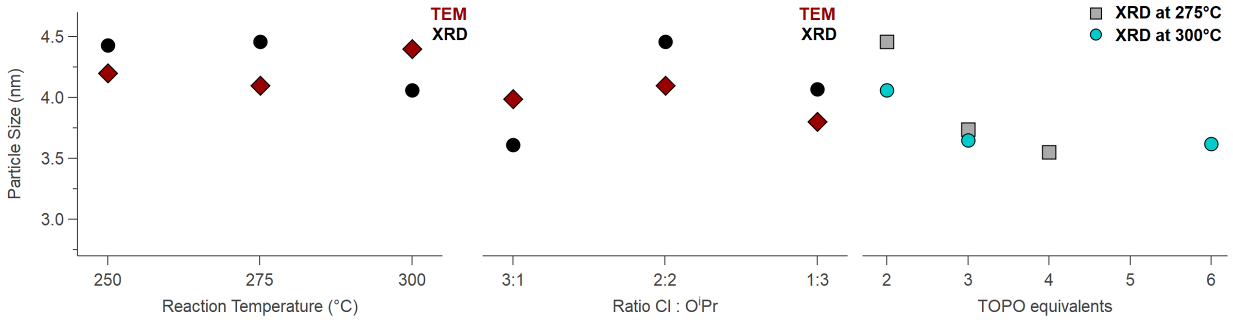

Figure S27: Dependence of crystal size of the ZrO_2 NCs calculated from XRD patterns in Figure S25 and BF TEM images when different reaction conditions were investigated.

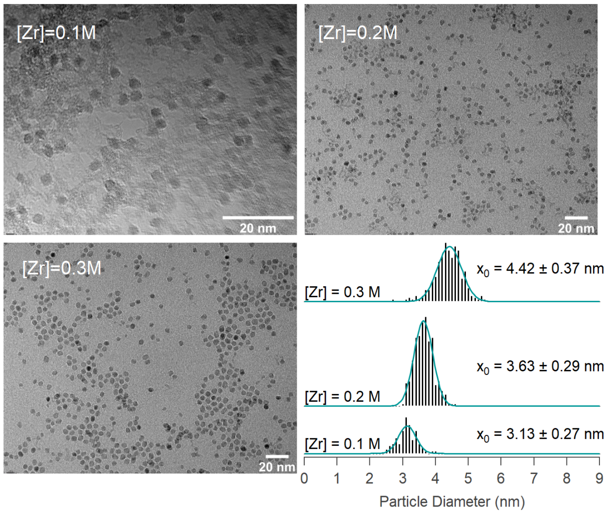

Figure S28: BF TEM images and size distribution of the ZrO_2 nanocrystals obtained from pure TOPO at 340 °C with different metal concentrations. The histograms are based on more than 100 nanoparticles.

References

(S1) De Keukeleere, K.; Coucke, S.; De Canck, E.; Van Der Voort, P.; Delpech, F.; Coppel, Y.; Hens, Z.; Van Driessche, I.; Owen, J. S.; De Roo, J. Stabilization of Colloidal Ti, Zr, and Hf Oxide Nanocrystals by Protonated Tri-n-octylphosphine Oxide (TOPO) and Its Decomposition Products. *Chemistry of Materials* **2017**, *29*, 10233–10242.

(S2) Bradley, D.; Mehrotra, R.; Wardlaw, W. 330. Hafnium alkoxides. *Journal of the Chemical Society (Resumed)* **1953**, 1634–1636.

(S3) Dhaene, E.; Seno, C.; De Roo, J. Synthesis of zirconium(IV) and hafnium(IV) isopropoxide, *sec*-butoxide and *tert*-butoxide. *Dalton Transactions* **2024**, *53*, 11769–11777.

(S4) Ballirano, P.; Caminiti, R. Rietveld refinements on laboratory energy dispersive X-ray diffraction (EDXD) data. *Journal of Applied Crystallography* **2001**, *34*, 757–762.

(S5) Howard, C. J.; Kisi, E. H.; Roberts, R. B.; Hill, R. J. Neutron diffraction studies of phase transformations between tetragonal and orthorhombic zirconia in magnesia-partially-stabilized zirconia. *Journal of the American Ceramic Society* **1990**, *73*, 2828–2833.
