# Supplementary material for: From Kinetics to Molecular-Level Insights into Group 4 Metal Oxide Nanocrystal Synthesis
Source: ACS Mater Au. 2025 May 29;5(4):709–17. doi: 10.1021/acsmaterialsau.5c00032 (PMC12257421; doi:10.1021/acsmaterialsau.5c00032)
Supplement: Supplementary file 2 [file mg5c00032_si_002.zip › Files Copasi/Readme.pdf]

Folders containing the input files (as .txt) and the Copasi files (.cps) for the data sets in Figure 6 and Figure S14. For each .cps there is a .txt file labeled with the same metal and concentration.

The .cps files contain the set of reactions used to fit the data. The initial concentrations of the species are gives as input (Model > Biochemical > Species) and to each reaction one k is assigned (Tasks > Parameter Estimation).

Select in Tasks > Parameter Estimation > Experimental Data the corresponding input file. Choose *Time Course* as Experiment Type and assign to OiPr the transient concentration of the species denoted by F (in the program is defined as the sum of the isopropoxide groups (Model > Biochemical > Species F)), as shown in the following panel:

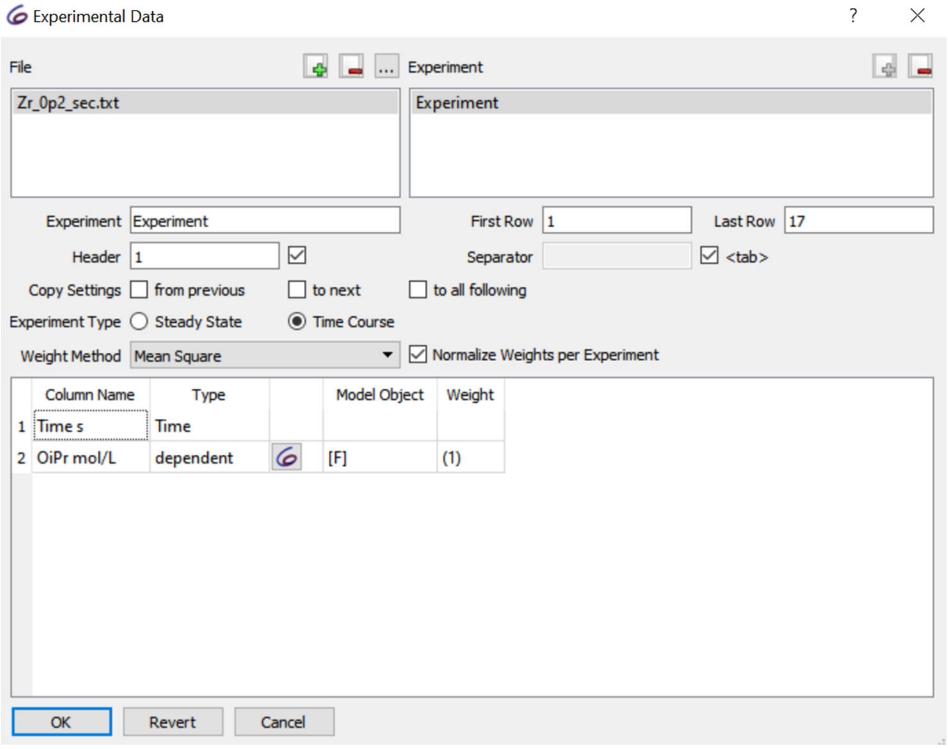

As output after the calculation is run, a plot of the concentration of the isopropoxide (labeled as [F]) as a function of the time is created, together with the determined k values (Tasks > Parameter Estimation > Result > Parameters).
